# Identification and Characterization of Oxidative Stress and Endoplasmic Reticulum Stress-Related Genes in Esophageal Cancer

**DOI:** 10.7150/jca.104376

**Published:** 2025-03-21

**Authors:** Xiaoxu Li, Juntao Lu, Yan Zhao, Wei Guo

**Affiliations:** 1 Department of Radiation Oncology, the Fourth Hospital of Hebei Medical University, Shijiazhuang, Hebei, China.; 2 Laboratory of Pathology, Hebei Cancer Institute, the Fourth Hospital of Hebei Medical University, Shijiazhuang, Hebei, China.; 3 Department of Oncology, the Fourth Hospital of Hebei Medical University, Shijiazhuang, Hebei, China.

**Keywords:** Esophageal cancer, Oxidative stress, Endoplasmic reticulum stress, TFRC

## Abstract

Increasing evidence highlights the critical roles of oxidative stress and endoplasmic reticulum (ER) stress in tumor initiation and progression. However, the specific functions of related genes in esophageal cancer (ESCA) remain poorly understood. To investigate the impact of oxidative and ER stress on ESCA, this study analyzed the TCGA and GEO databases to identify 12 oxidative stress- and ER stress-related differentially expressed genes (OERDEGs). Pathway analysis revealed significant enrichment in critical processes such as PRC2-mediated methylation, oxidative stress-induced senescence, and NOTCH signaling. A novel LASSO regression model was developed to link gene expression with clinical prognosis, and the model was validated through ROC and Cox regression analyses. Four OERDEGs (CDKN3, PINK1, SPP1, and TFRC) were identified as key biomarkers for ESCA prognosis. Notably, TFRC expression was significantly upregulated in ESCA cells under both oxidative and ER stress conditions, in a dose- and time-dependent manner. Functional assays confirmed that TFRC promotes cell proliferation, migration, and invasion by regulating the HIF-1α and NOTCH1 signaling pathways. This study elucidates the complex interplay between oxidative/ER stress and ESCA progression and highlights the innovative application of bioinformatics to identify potential biomarkers for early diagnosis and therapeutic strategies. Targeting TFRC, in particular, may offer a novel approach to improving ESCA treatment and enhancing patient prognosis.

## Introduction

ESCA is the seventh most prevalent cancer globally and ranks sixth in cancer-related mortality 1. It primarily includes two histological subtypes: esophageal squamous cell cancer (ESCC) and esophageal adenocarcinoma (EAC). Despite advances in multimodal treatments, such as surgery and chemoradiotherapy, ESCA is still associated with poor prognosis and low five-year survival rates 2, 3. The limited efficacy of existing targeted therapies emphasizes the urgent need for novel biomarkers to enhance diagnosis, prognosis, and treatment outcomes.

Oxidative stress and ER stress are key factors in various physiological and pathological processes, and their roles in carcinogenesis are well established. Oxidative stress results from an imbalance between reactive oxygen species (ROS) production and the cellular antioxidant defense mechanisms, leading to DNA, protein, and lipid damage 4. Excessive ROS generation disrupts cellular functions, promotes genetic mutations and contributes to tumorigenesis 5, 6. Elevated ROS levels in cancer cells have been shown to enhance tumor growth and malignancy by further disrupting cellular signaling and promoting cancer progression 7-9.

ER stress occurs when there is an accumulation of misfolded or unfolded proteins within the ER, activating the unfolded protein response (UPR) to restore homeostasis 10. High levels of ER stress and UPR activation have been observed in various cancers, including lung, breast, and colorectal cancers, which have been linked to tumorigenesis, proliferation, metastasis, and treatment resistance 11-13. Recent studies have revealed that oxidative stress and ER stress are interconnected and contribute to cancer through mechanisms such as epithelial-mesenchymal transition (EMT), angiogenesis, and modulation of the tumor microenvironment 14-17.

In ESCA, oxidative stress and ER stress are recognized as important contributors to tumorigenesis and progression 18, 19. However, the specific genes involved in these stress responses and their underlying molecular mechanisms in ESCA remain largely unexplored. To address this knowledge gap, our study employed bioinformatics approaches to identify and characterize oxidative stress and ER stress-related genes in ESCA.

By integrating transcriptome data from TCGA and GEO databases, we identified 12 OERDEGs that play significant roles in ESCA pathogenesis. Our comprehensive analyses included Gene Ontology (GO) and Kyoto Encyclopedia of Genes and Genomes (KEGG) pathway enrichment, Gene Set Enrichment Analysis (GSEA), Gene Set Variation Analysis (GSVA), protein-protein interaction (PPI) network construction, and transcriptional regulation assessments. To develop a prognostic model, we applied least absolute shrinkage and selection operator (LASSO) regression and validated the model through receiver operating characteristic (ROC) curve analysis and univariate and multivariate Cox regression. Additionally, qRT-PCR was performed to validate the expression of these OERDEGs in samples from 105 ESCA patients, providing insights into their prognostic significance. Among these, TFRC was particularly investigated for its potential biological functions in ESCA cells, revealing its role in cell proliferation, migration, and invasion.

## Materials and methods

### Data Acquisition

RNA sequencing data in fragments per kilobase per million (FPKM) format were obtained from 162 ESCA tissues and 11 normal tissues from the TCGA database using the “TCGAbiolinks” R package. Clinical data were sourced from the UCSC Xena database. Additionally, the transcriptome data and clinical information from the GSE20347 dataset were retrieved from the GEO database using the “GEOquery” R package. This dataset included microarray gene expression profiles for 17 ESCA tissues and their corresponding paired paracancer tissues.

To identify ER stress-related genes (ERGs), we initially compiled a list of 342 ERGs from the MSigDB database and supplemented it with an additional 785 ERGs from a previous study 20. After merging these lists and removing duplicates, we identified 871 ERGs. Similarly, oxidative stress-related genes (ORGs) were compiled, starting with 727 ORGs from MSigDB and incorporating an additional 1,119 ORGs from prior research, resulting in a total of 1,326 ORGs 21. By combining the ERGs and ORGs, we identified 388 oxidative stress and ER stress-related genes (OERGs) (Table S1).

### Analysis of differential expression

The gene expression profiles of both the cancer and normal groups in the TCGA-ESCA dataset were subjected to analysis to detect differentially expressed genes (DEGs) using the R package “limma”. To visualize the differences in expression, we created volcano plots using the R package "ggplot2". Similarly, in the normalized GSE20347 dataset, we compared expression profiles between the cancer and normal groups to identify DEGs, employing the “limma” package. For visual representation of gene expression in the TCGA-ESCA dataset, we utilized the R package "pheatmap" to generate heatmaps.

### GO/KEGG enrichment analysis

We used the R package “clusterProfiler” to conduct GO annotation analysis of the 12 OERDEGs. Significance was assigned to categories with a p-value below 0.05 and a false discovery rate (FDR) q-value below 0.2. The Benjamini-Hochberg (BH) procedure was utilized to control FDR by adjusting P values.

### GSEA

We performed GSEA on both the cancer and normal groups within the TCGA-ESCA dataset, employing the R package “clusterProfiler”. Each analysis included 1,000 gene set permutations, utilizing “c2.cp.v7.2.symbols” from MSigDB Collections as the reference gene collection. The threshold value of statistical significance was set as P.adj < 0.05 and FDR q-value < 0.2. The BH procedure was used to adjust P values and control FDR.

### GSVA

To conduct GSVA between the cancer and normal groups in the TCGA-ESCA dataset, we obtained the “h.all.v7.4.symbols.gmt” gene set collection from the MSigDB database. A significance level of P < 0.05 was used to identify significantly associated genes.

### PPI analysis

To elucidate interactions among OERDEGs, we created a PPI network using the STRING database with interactions having a score greater than 0.4 and visualized it using Cytoscape software. Furthermore, GeneMANIA was employed to predict genes with similar functions related to the OERDEGs and to create an interaction network.

### Regulation Network

We used the DGIdb database to predict potential small molecule compounds or drugs that interact with OERDEGs, and we visualized the interaction network using Cytoscape software. To identify transcription factors (TFs) binding to OERDEGs, we utilized the CHIPBase and hTFtarget databases and then visualized the resulting mRNA-TF interaction network with Cytoscape software.

### Construction of prognostic model

We utilized LASSO regression analysis to construct a prognostic model for OERDEGs in ESCA, and determined the penalty regularization parameter lambda through a 10-fold cross-validation procedure. The risk score was calculated using the following formula.



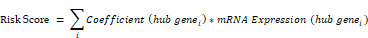



### ROC curve

We generated ROC curves using the “survivalROC” package and subsequently calculated the area under the curve (AUC) to assess the predictive capacity of OERDEGs' expression for the survival of ESCA patients. In time-dependent ROC curves, an AUC above 0.5 indicates that the gene expression promotes the event, while an AUC below 0.5 suggests inhibition. A greater deviation from the line of equality corresponds to higher diagnostic accuracy in the respective direction. By plotting time-dependent ROC curves, we explored the relationship between the expression of OERDEGs and the prognosis of ESCA patients.

### Cox analysis

We evaluated the impact of OERDEGs expression on ESCA prognosis through multivariate Cox regression analysis and presented the results using forest plots. The threshold for the P value was set at 0.1. Nomograms were constructed using the independent prognostic factors to provide personalized survival probability estimates for 1, 3, and 5 years. To further evaluate the predictive accuracy and discrimination ability of the nomograms, we used calibration curves. The RMS package was employed to generate nomograms and calibration curves. Decision curve analysis (DCA) was conducted using the “ggDCA” package to assess the predictive accuracy of the nomogram.

### Patients and tissue specimens

This study used tissue samples from 105 patients with ESCA, diagnosed based on histopathological examination. These patients underwent surgical resection at the Fourth Hospital of Hebei Medical University between 2015 and 2019. Exclusion criteria included patients who had received neoadjuvant chemotherapy or radiotherapy prior to surgery, those with a history of other malignancies, severe infections, autoimmune diseases, or other chronic conditions, as well as those with incomplete clinical data. Cancer tissues and matched adjacent normal tissues were collected during surgery and immediately frozen in liquid nitrogen after resection.

### RNA isolation and quantitative real-time polymerase chain reaction (qRT-PCR) assay

Total RNA was extracted from tissue samples or cells utilizing the TRIzol reagent (Invitrogen, Carlsbad, CA, USA). The extracted RNA was reverse transcribed into cDNA with the Transcriptor First Strand cDNA Synthesis Kit (Roche, Basel, Switzerland) following the manufacturer's protocol. The StepOnePlus Real-Time PCR System (Applied Biosystems) was used for qRT-PCR assays with SYBR Green Real-Time PCR Master Mix (Solarbio, Beijing, China). Expression levels were normalized using GAPDH as an endogenous control, and relative expression was determined using the 2^-ΔΔCt^ method. The primer sequences utilized in the qRT-PCR analysis can be found in Table S2.

### Cell culture

Human ESCC cell lines (KYSE150 and TE1), an EAC cell line (OE33), and a human normal esophageal epithelial cell line (HEEC) were obtained from the China Center for Type Culture Collection (CCTCC, Wuhan, China). The ESCC and EAC cell lines were cultured in RPMI 1640 medium (Gibco, Invitrogen, Life Technologies, Germany) supplemented with 10% heat-inactivated fetal bovine serum (FBS) (Invitrogen, Carlsbad, CA, USA), at 37°C in a humidified atmosphere containing 5% CO_2_. HEEC cells were cultured according to the manufacturer's instructions.

### Cell transfection

GenePharma (Shanghai, China) prepared the TFRC plasmid, while small interfering RNAs (siRNAs) targeting TFRC were also obtained from the same supplier. Transfection of cells with plasmids or siRNAs was carried out using Lipofectamine 2000 (Invitrogen) following the manufacturer's instructions.

### Cell proliferation assay

Cellular proliferation capacity was assessed using MTS assays and colony formation assays. The CellTiter 96® Aqueous One Solution Cell Proliferation Assay kit (Promega, Madison, WI, USA) was used to perform MTS assays. Transfected cells were seeded in 96-well plates at a density of 1×10^3^ cells per well, with each group replicated six times. Cell viability was determined by measuring the absorbance of each well at 490nm using a microplate reader after adding 20μL of MTS reagent (500 μg/mL) to each well at various time intervals and incubating for 2 hours. For colony formation assays, 3×10^3^ transfected cells were plated in each well of 6-well plates and incubated for 5-7 days until visible colonies formed. Colonies containing 50 or more cells were counted after fixation with 4% paraformaldehyde and staining with 0.1% crystal violet.

### Transwell migration and invasion assays

For transwell migration assays, we seeded 1×10^5^ cells suspended in 200μL of serum-free RPMI 1640 medium into the top chamber (Corning Costar, Corning, NY, USA), while the bottom chamber contained 600μL of medium with 10% FBS. After an incubation of 12-24 hours, cells that had migrated to the lower surface of the membrane were fixed with 4% paraformaldehyde and stained with crystal violet. The migration rate was determined by manually counting the number of cells in at least five different fields using an inverted microscope at 20× magnification. For cell invasion assays, we followed a similar protocol to the cell migration assay, with the exception that the top chamber was coated with 50 µL Matrigel (Corning) and incubated for 2 hours.

### Western Blot assay

Total protein was extracted following the manufacturer's protocol using RIPA lysis buffer containing PMSF (Solarbio). Protein concentrations were measured using the BCA Protein Assay Kit (Multi-Science, Hangzhou, China). Equal amounts of protein were subjected to 10% polyacrylamide gel electrophoresis and transferred onto PVDF membranes (Millipore, Sigma, Burlington, MA, USA). After blocking with 5% skim milk, the membranes were incubated overnight at 4°C with specific primary antibodies, followed by a one-hour incubation with secondary antibodies at room temperature. Protein bands were visualized using an enhanced chemiluminescence reagent (Multi Sciences) and detected with the Chemi XT4 system (Syngene). The primary antibodies used are listed in Table S3.

### Statistical analysis

We conducted all statistical analyses using two software programs, R-4.1.2 and SPSS 25.0. Continuous variables were presented as the mean ± standard deviation. To compare continuous variables between two groups, we used the Wilcoxon rank sum test, while the independent Student t-test was employed for normally distributed variables. For comparisons among three or more groups, we used the Kruskal-Wallis test. Categorical variables were assessed for statistical significance in pairwise comparisons using either the chi-square test or Fisher's exact test. LASSO regression analysis was performed using the R package “glmnet”. Kaplan-Meier analyses were used to generate overall survival curves, with statistical significance determined by the log-rank test for P values. Unless otherwise indicated, correlation coefficients between different molecules were calculated using Spearman correlation analysis. All P-values were two-sided, and statistical significance was set at a level less than 0.05.

## Results

### Differential expression analysis

**Figure 1:**
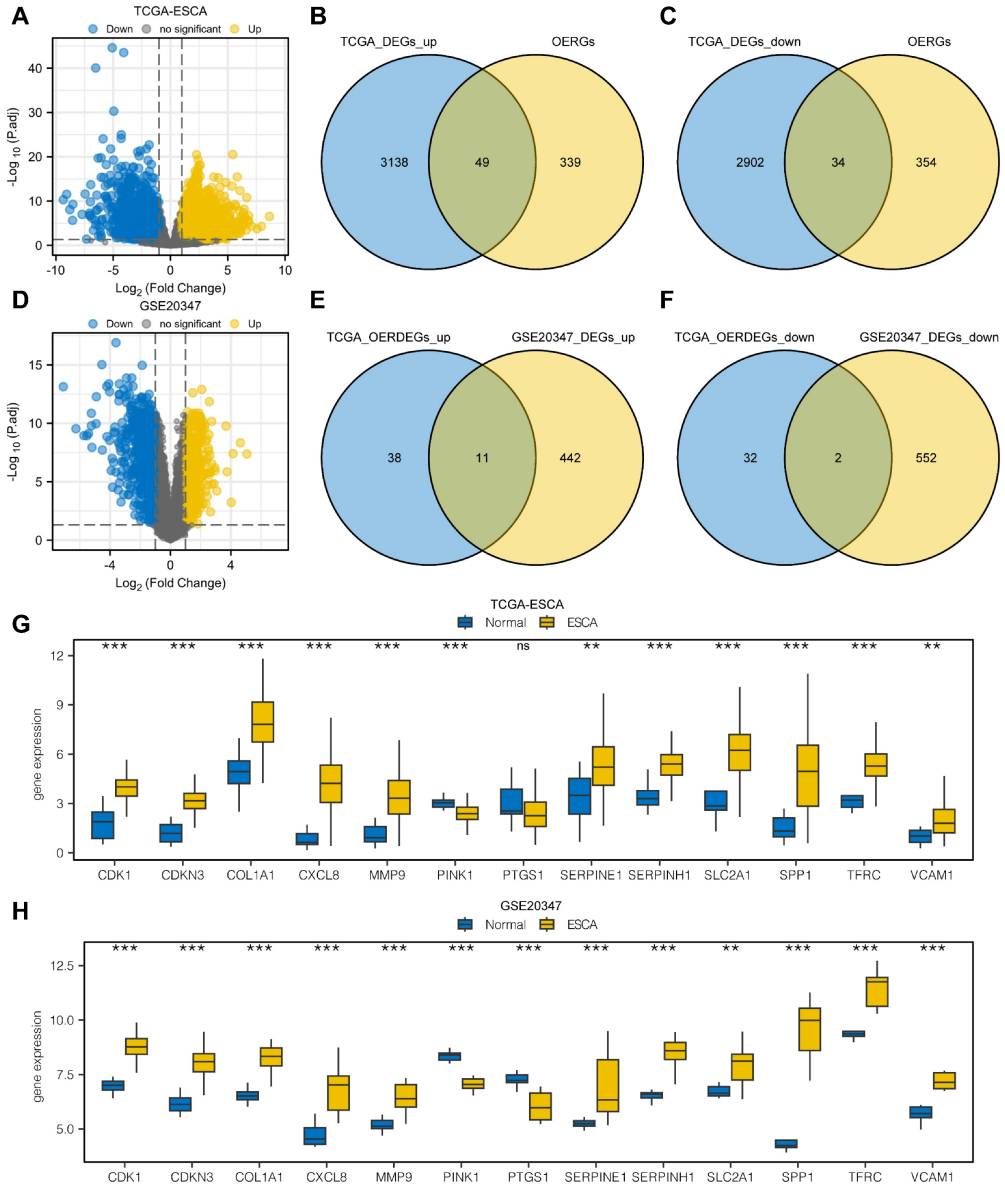
** Analysis of differentially expressed genes. A** The volcano plot illustrates the results of the differential expression analysis conducted between the cancer group (ESCA) and normal group (Normal) using the dataset from The Cancer Genome Atlas (TCGA-ESCA). **B** The Venn diagram shows the overlap between the upregulated differentially expressed genes (DEGs) and oxidative stress and ER stress-related genes (OERGs) obtained from the TCGA-ESCA dataset. **C** The Venn diagram presents the intersection between the downregulated DEGs and OERGs obtained from the TCGA-ESCA dataset. **D** The Volcano plot represents the findings of the differential expression analysis between the cancer group (ESCA) and normal group (Normal) in the Gene Expression Omnibus (GEO) dataset GSE20347. **E** The Venn diagram displays the overlap between the upregulated oxidative stress and ER stress-related differentially expressed genes (OERDEGs) obtained from the TCGA-ESCA dataset and the upregulated DEGs obtained from the GSE20347 dataset. **F** The Venn diagram shows the intersection between the downregulated OERDEGs obtained from the TCGA-ESCA dataset and the downregulated DEGs obtained from the GSE20347 dataset. **G-H** Comparative graphical presentations depict the grouping of OERDEGs in both the TCGA-ESCA and GSE20347 datasets. ns represents P > 0.05, * P < 0.05, ** P < 0.01, *** P < 0.001.

From the differential expression analysis of the TCGA-ESCA dataset, we identified 6,136 DEGs that met the criteria of |logFC| > 1 and P.adj < 0.05 from a total of 56,494 genes. Among these, 3,190 genes were upregulated in the cancer group, and 2,946 genes were downregulated. The DEGs were visualized using a volcano map (Fig. 1A). We further conducted intersections between the upregulated DEGs and OERGs, resulting in 49 genes shown in the Venn diagram (Fig. 1B). Similarly, for the downregulated DEGs, the intersection with OERGs yielded 34 genes, as depicted in the Venn diagram (Fig. 1C). Comparing the cancer and normal groups in the GSE20347 dataset, we created a volcano plot to visualize the results of the differential expression analysis (Fig. 1D). In the ESCA tissues, we identified 453 upregulated (logFC > 1 and P.adj < 0.05) and 554 downregulated DEGs (logFC < -1 and P.adj < 0.05). We then intersected the upregulated OERDEGs identified from the TCGA dataset with the upregulated DEGs from GSE20347, as well as the downregulated DEGs, resulting in 13 OERDEGs that exhibited consistent differential expression patterns in both datasets (Fig. 1E, 1F). Detailed information regarding the names and expression differences of these 13 OERDEGs can be found in Table S4 and S5. Subsequently, the expression patterns of the 13 OERDEGs were analyzed in the TCGA-ESCA and GSE 20347 datasets, respectively, using grouped comparison plots (Fig. 1G, 1H). Remarkably, a total of 12 genes (CDK1, CDKN3, COL1A1, CXCL8, MMP9, PINK1, SERPINE1, SERPINH1, SLC2A1, SPP1, TFRC, and VCAM1) exhibited consistent results in both the TCGA-ESCA dataset and the GSE20347 dataset, highlighting their potential significance as key OERDEGs.

### Heat map and GO/KEGG analysis

**Figure 2:**
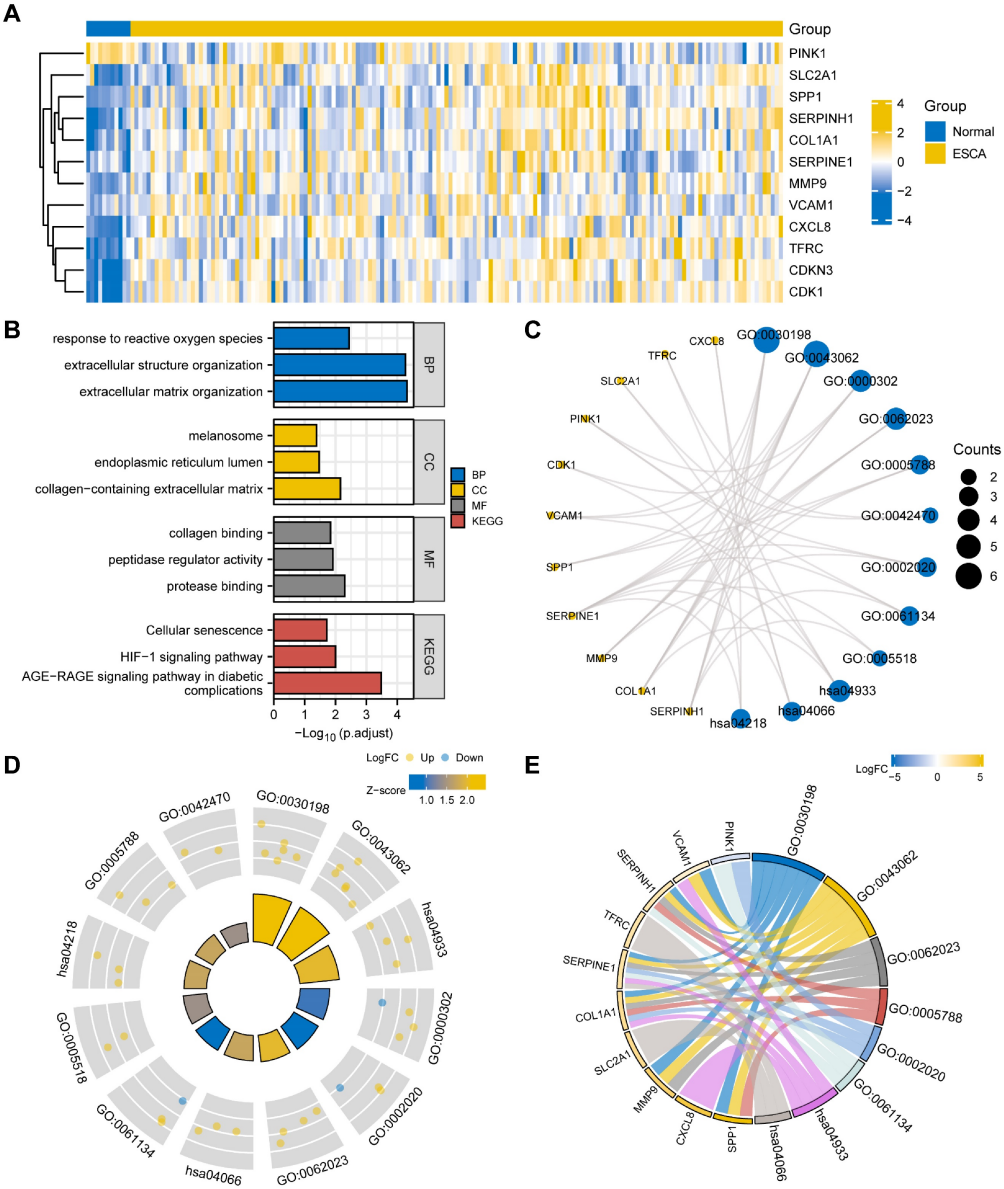
** Heat map and GO/KEGG analysis of OERDEGs. A** The heat map illustrates the expression of OERDEGs in the TCGA-ESCA dataset. **B** The histogram depicts the results of the GO/KEGG enrichment analysis for OERDEGs. **C** The network divergence plot displays the results of the GO/KEGG enrichment analysis for OERDEGs. **D** The chord plot visualizes the results of the joint logFC GO/KEGG enrichment analysis for OERDEGs. **E** The circular plot provides an overview of the GO/KEGG enrichment analysis results for OERDEGs. The screening criteria for inclusion in the GO/KEGG enrichment entries were P < 0.05 and FDR value (q.value) < 0.2.

To start, we generated a heatmap illustrating the expression levels of the 12 OERDEGs (CDK1, CDKN3, COL1A1, CXCL8, MMP9, PINK1, SERPINE1, SERPINH1, SLC2A1, SPP1, TFRC, VCAM1) in the TCGA-ESCA dataset (Fig. 2A). To understand the potential functions of these OERDEGs, we conducted KEGG enrichment analysis to reveal their involvement in various biological processes (BPs), cellular components (CCs), and molecular functions (MFs). These functions included extracellular matrix organization (GO: 0030198), extracellular structure organization (GO: 0043062), response to reactive oxygen species (GO: 0000302), collagen-containing extracellular matrix (GO: 006202), endoplasmic reticulum lumen (GO: 000578), melanosome (GO: 0042470), protease binding (GO: 0002020), peptidase regulator activity (GO: 0061134), and collagen binding (GO: 0005518) (Table S6). KEGG pathway analysis, shown in Fig. 2B and 2C, highlighted significant signaling pathways, including the AGE-RAGE signaling pathway in diabetic complications (hsa04933), HIF-1 signaling pathway (hsa04066), and Cellular senescence (hsa04218). To provide additional insights, we integrated the results of GO/KEGG analysis with the logFC values for enrichment analysis. Specifically, we computed the Z-score for each OERDEG based on its logFC value obtained from the differential analysis between cancer and normal groups in the TCGA-ESCA dataset. The findings suggested that extracellular matrix organization (GO: 0030198) and extracellular structure organization (GO: 0043062) were significantly upregulated BPs. Additionally, the AGE-RAGE signaling pathway in diabetic complications (hsa04933) was a significantly upregulated KEGG pathway (Fig. 2D, 2E).

### GSEA and GSVA

**Figure 3:**
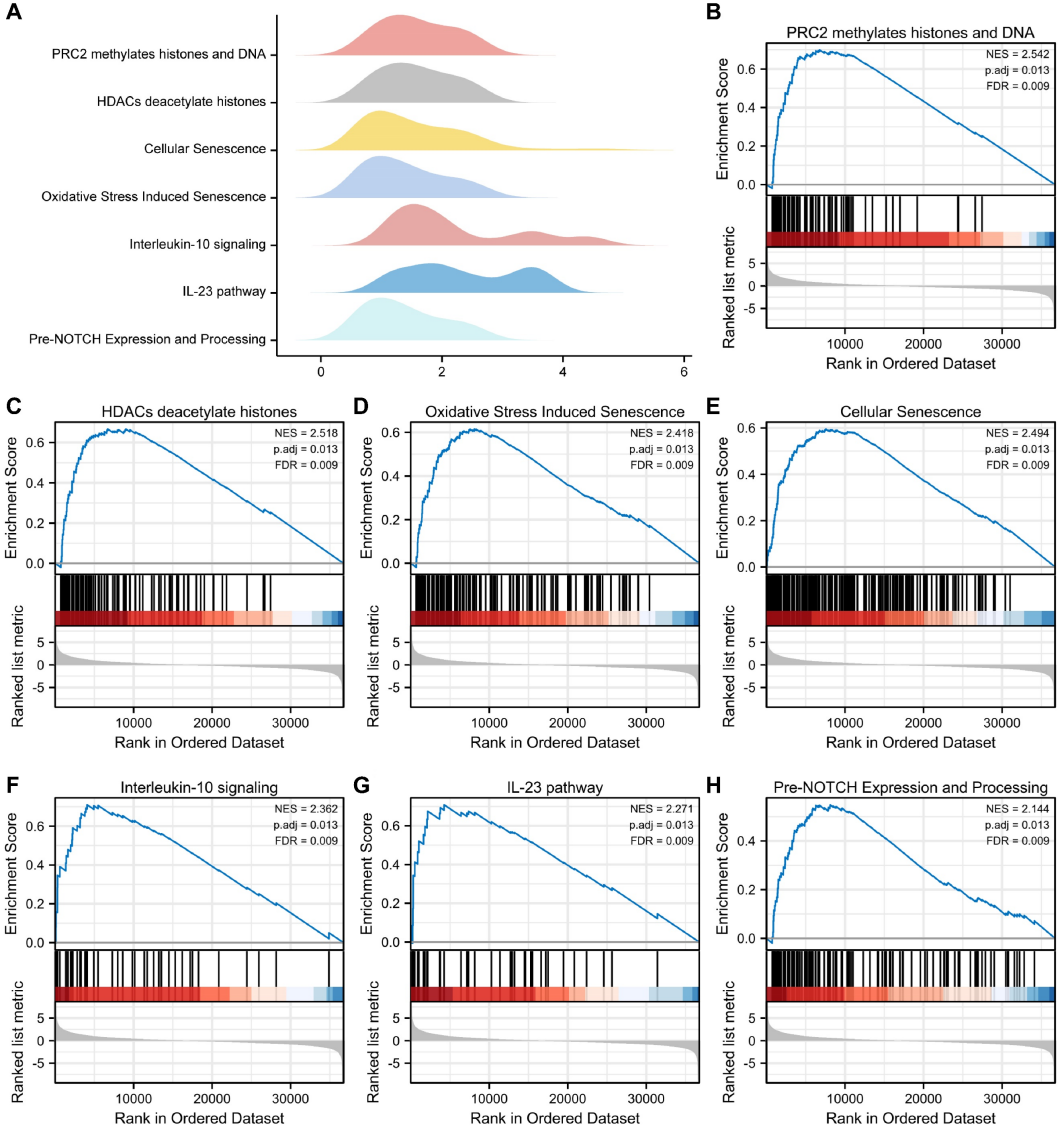
** GSEA. A** The main biological features derived from GSEA of the TCGA-ESCA dataset. **B-H** In the TCGA-ESCA dataset, genes exhibited significant enrichment in several pathways, including PRC2 methylates histones and DNA (B), HDACs deacetylate histones (C), Oxidative Stress Induced Senescence (D), Cellular Senescence (E), Interleukin-10 signaling (F), IL-23 pathway (G), Pre-NOTCH Expression and Processing (H), and others. The criteria for significant enrichment in the GSEA analysis were P.adj < 0.05 and FDR value (q.value) < 0.2.

To examine the differential effects of these genes on cancer and normal groups, we conducted GSEA to investigate the association between OERDEG expression levels and corresponding BPs, CCs, and MFs in the TCGA-ESCA dataset. As illustrated in Fig. 3A-H and detailed in Table S7, these genes exhibited significant enrichment in pathways, including PRC2-mediated methylation of histones and DNA, HDACs deacetylate histones, Oxidative Stress Induced Senescence, Cellular Senescence, Interleukin-10 signaling, IL-23 pathway, pre-NOTCH expression and processing, among others.

**Figure 4:**
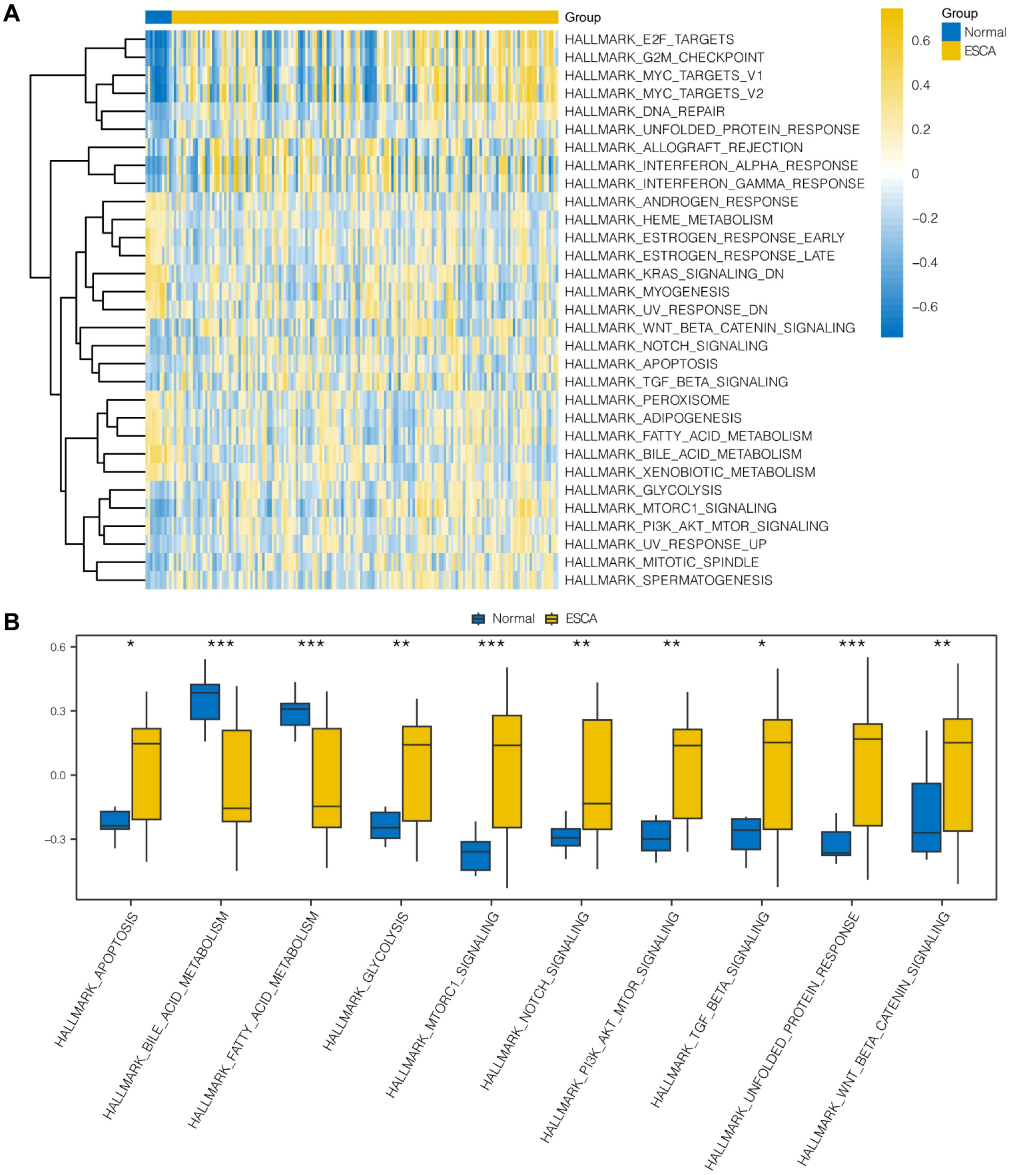
** GSVA. A** The heat map represents functional scores generated through GSVA in the TCGA-ESCA dataset. **B** Grouped comparison plots displays enriched pathways with notably significant differences derived identified through GSVA in the TCGA-ESCA dataset. * P < 0.05, ** P < 0.01, *** P < 0.001.

To explore the differences in hallmark gene sets between the cancer and normal groups in ESCA, we conducted GSVA to assess the expression of OERDEGs in the TCGA-ESCA dataset. As shown in Fig. 4A and Table S8, there were 31 hallmark gene sets that showed significant differences (P < 0.05) between cancer and normal groups in ESCA. We specifically selected 10 hallmark gene sets that showed highly significant differences (P < 0.001) or played crucial roles in tumor progression, presenting group comparisons in Fig. 4B. Our examination of the TCGA-ESCA dataset demonstrated significant enrichment of genes associated with various pathways, including hallmark apoptosis, PI3K-AKT-mTOR signaling, fatty acid metabolism, glycolysis, and NOTCH signaling pathways, among others.

### PPI network

**Figure 5:**
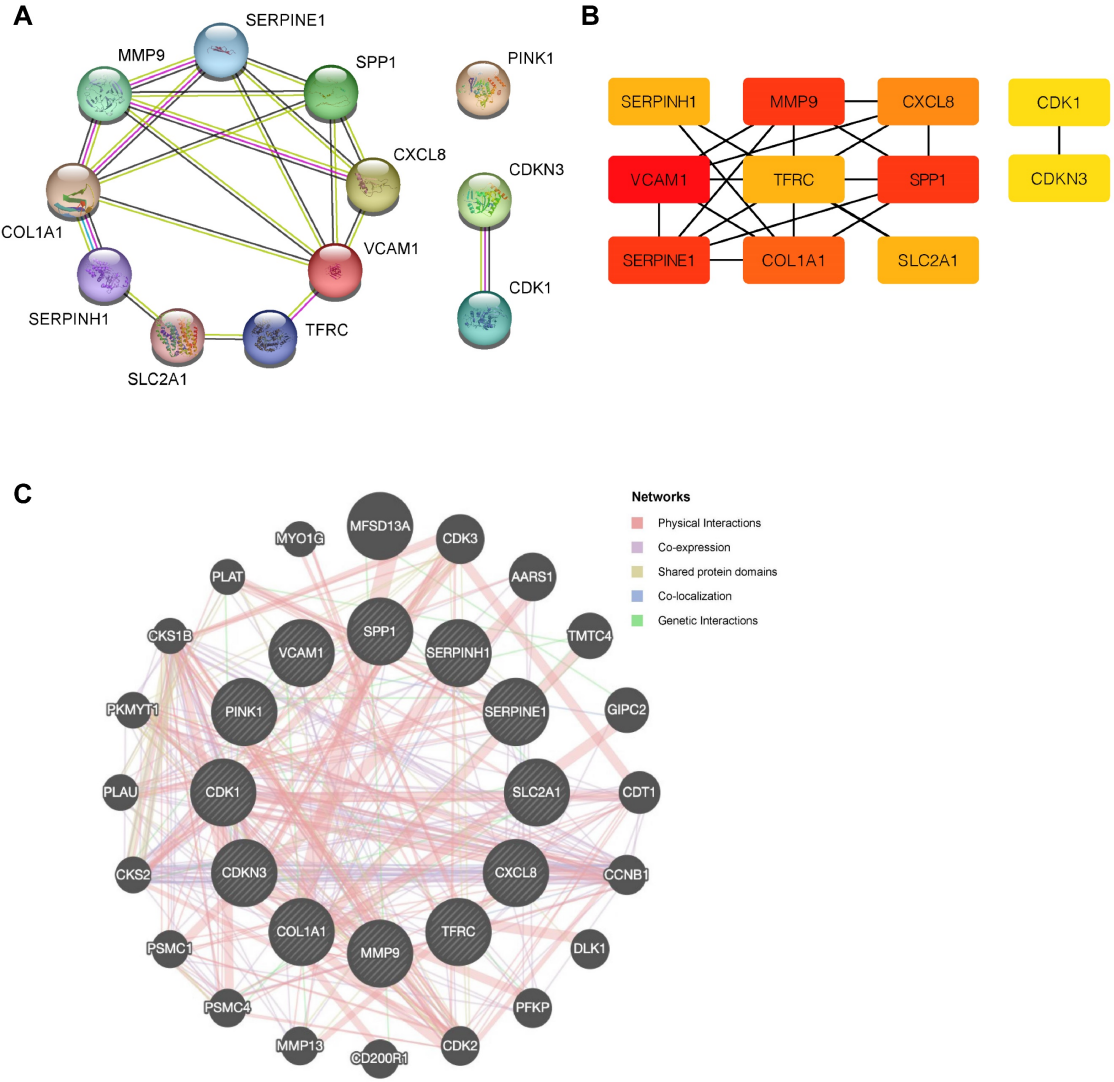
** PPI network. A** The PPI network of OERDEGs. **B** The MCC algorithm was used to identify hub genes in the PPI network. The color spectrum, ranging from yellow to red, signifies the gradual increase in scores. **C** The association network of functionally similar genes among OERDEGs predicted by GeneMANIA. The black circles with white slashes represent input OERDEGs, while other solid black circles denote predicted functionally similar genes.

To explore potential protein-protein interactions among OERDEGs, we created a PPI network with a minimum interaction score of 0.4, indicating medium confidence. As shown in Fig. 5A, all 11 OERDEGs, except PINK1, interacted with at least one other OERDEG, with VCAM1 exhibiting the highest number of interactions. Additionally, we applied the Maximal Clique Centrality (MCC) algorithm to calculate scores for each gene node. OERDEGs were color-coded from red to yellow according to their scores, with VCAM1 having the highest MCC score (Fig. 5B). Detailed gene score levels can be found in Table S9. Furthermore, we constructed an association network between the 12 OERDEGs and other genes sharing similar biological functions using GeneMANIA to predict interactions, co-expression, and co-localization (Fig. 5C).

### Regulation network

**Figure 6:**
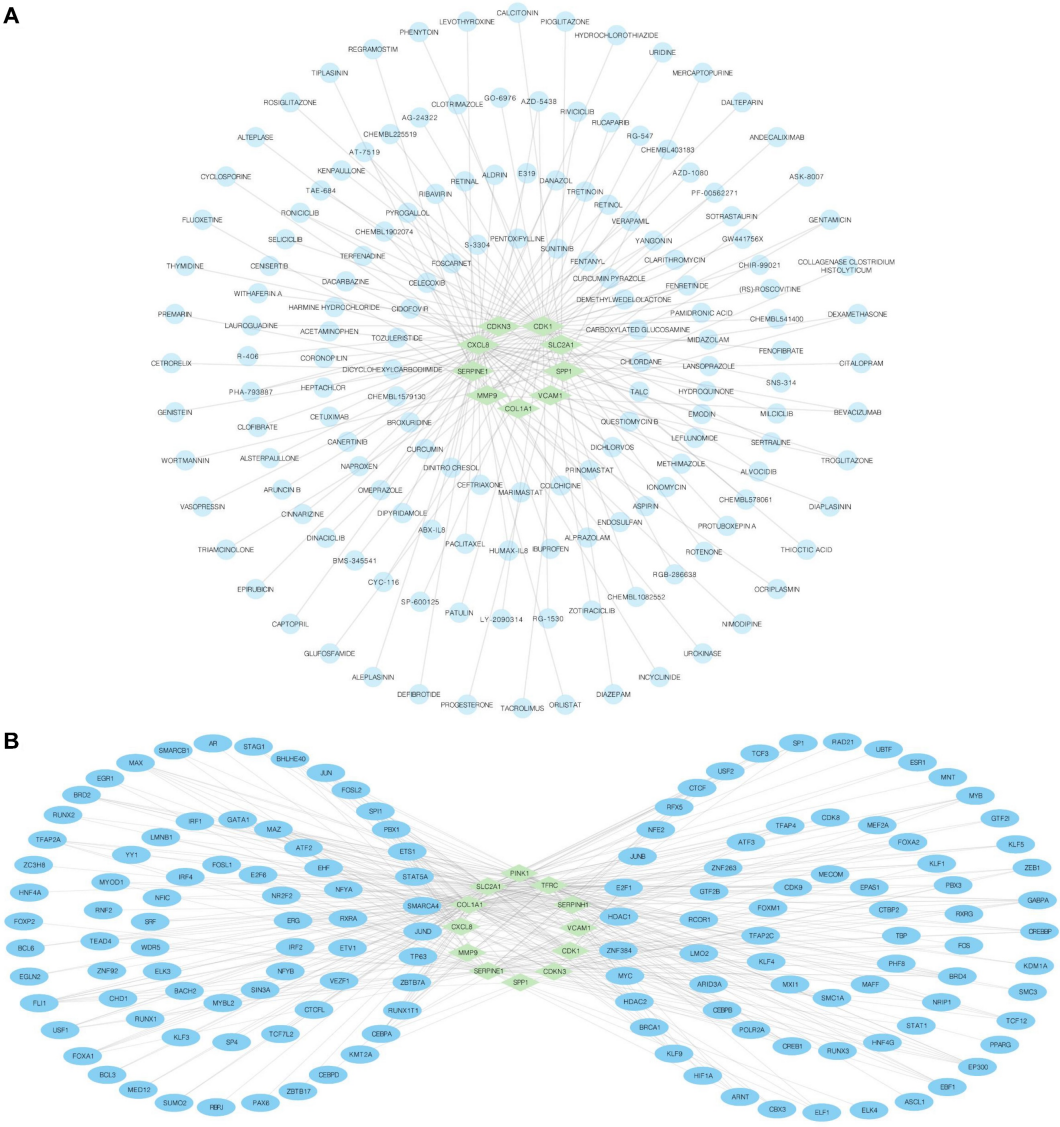
** mRNA-drug and mRNA-TF networks. A** The mRNA-drug regulatory network of OERDEGs, where the green diamond squares representing mRNAs and the blue dots symbolizing drugs. **B** The mRNA-TF regulatory network of OERDEGs, where the green diamond squares representing mRNA and the blue ovals denoting TFs.

We employed the DGIdb database to identify potential molecular compounds for OERDEGs. A total of 155 potential molecular compounds or drugs that matched 9 OERDEGs (CDK1, CDKN3, COL1A1, CXCL8, MMP9, SERPINE1, SLC2A1, SPP1, VCAM1) were found (Fig. 6A, Table S10). These compounds offer promising therapeutic options for ESCA and valuable insights into novel therapeutic strategies. Next, the CHIPBase and hTFtarget databases were utilized to identify TFs that bind to hub genes. The interaction results were intersected to gather data on the connections between the 12 OERDEGs and 142 TFs, forming the mRNA-TF interaction network (Fig. 6B, Table S11). These TF interactions with OERDEGs may play crucial regulatory roles in the pathogenesis of ESCA.

### LASSO model

**Figure 7:**
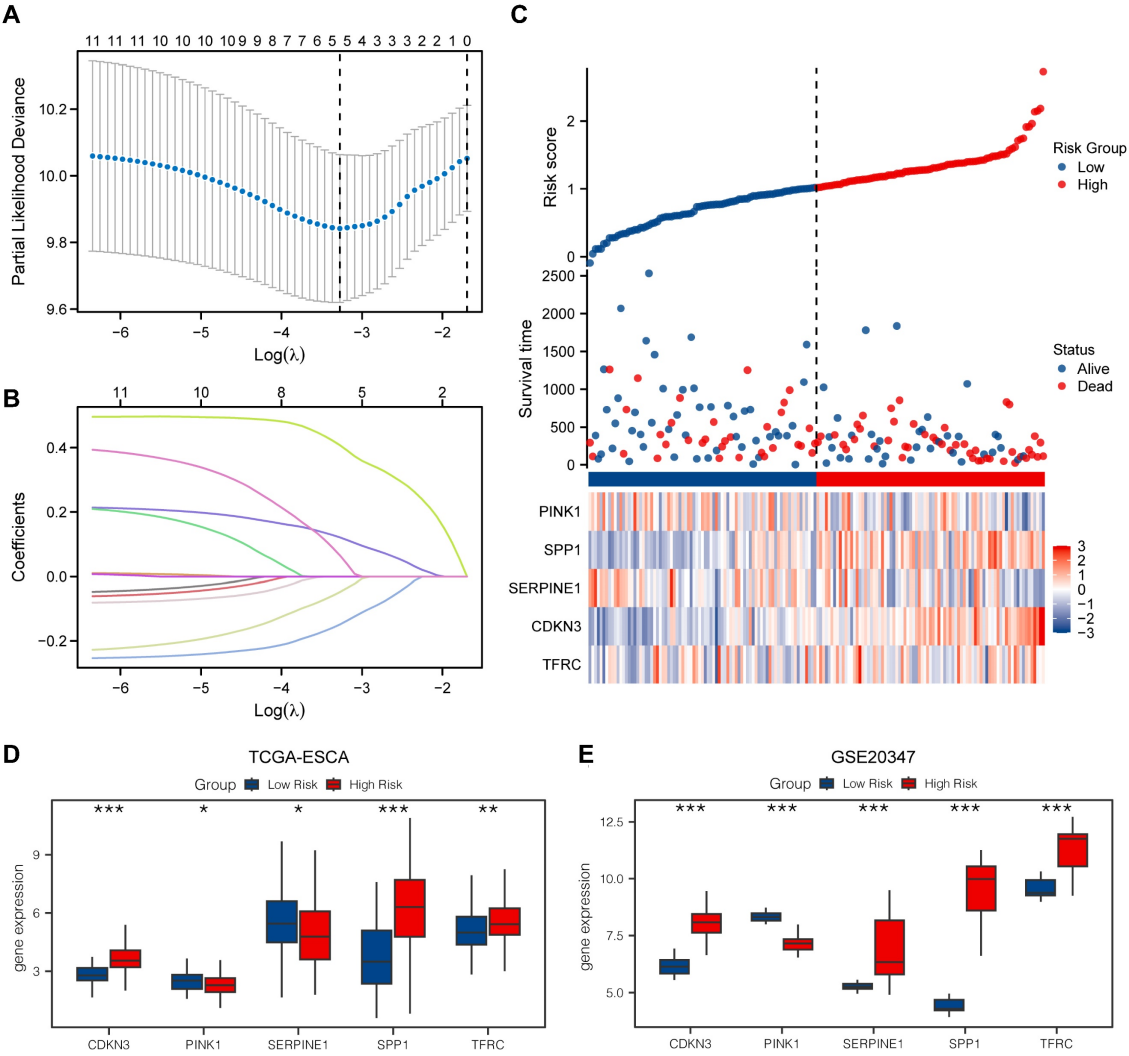
** Construction of a prognostic model for OERDEGs and differential genetic analysis of LASSO high- and low-risk groups.** A The plot of the LASSO regression prognostic model for OERDEGs. B-C The trajectory plots of variables for the LASSO regression diagnostic model (B), and risk factor plots (C). D-E The group comparison plots for OERDEGs in the TCGA-ESCA dataset (D), and the GSE20347 dataset (E).

We utilized the LASSO model to assess the prognostic value of the OERDEGs. The LASSO algorithm applies penalty terms to encourage sparse solutions, effectively reducing the coefficients of irrelevant or less important variables to zero. Consequently, some initially identified OERDEGs were excluded, resulting in a final model that comprises five OERDEGs (CDKN3, PINK1, SERPINE1, SPP1, TFRC). The results of the LASSO analysis for constructing prognostic models for these five OERDEGs are presented in Fig. 7A and 7B. Using the risk score formula, we determined the median value to divide the cancer group into two equal subgroups: the low-risk group and the high-risk group, as shown in Fig. 7C. To validate the LASSO prognostic model, we analyzed clinical data obtained from the TCGA-ESCA dataset (Table S12). Subsequently, the coefficients of the variables in the LASSO model were used to compute risk scores for the cancer group from the GSE20347 dataset. The cancer group was divided into high-risk and low-risk subgroups based on the risk score. Group comparisons were plotted based on the expression of each OERDEG between these subgroups in both the TCGA-ESCA dataset and the GSE20347 dataset. A statistically significant difference in expression was indicated by P < 0.05. As illustrated in Fig. 7D and 7E, the validation confirmed consistent results for four genes, namely CDKN3, PINK1, SPP1, and TFRC.

### ROC and clinical correlation analysis

**Figure 8:**
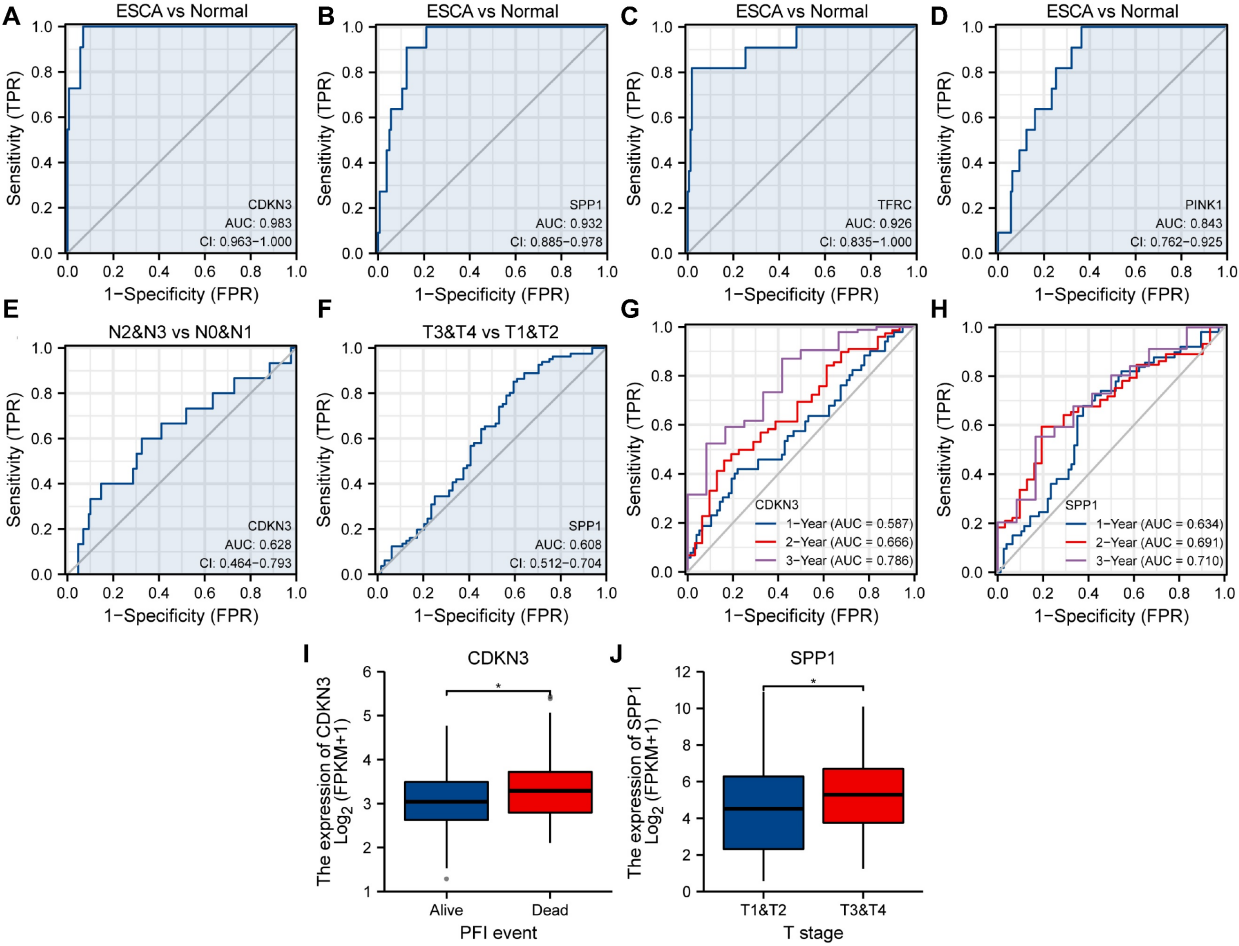
** ROC curves and clinical correlation analysis. A-D** ROC curves assessing CDKN3 (A), SPP1 (B), TFRC (C), and PINK1 (D) as predictors for ESCA versus Normal. **E** ROC curve examining CDKN3 as a predictor for N0 & N1 versus N2 & N3. **F** ROC curves evaluating SPP1 as a predictor for T1&T2 versus T3&T4. **G** 1-year, 2-year, and 3-year time-dependent ROC curves for CDKN3. **H** 1-year, 2-year, and 3-year time-dependent ROC curves for SPP1. **I** Subgroup comparison plots of CDKN3 in clinical correlation analysis of PFI.** J** Subgroup comparison plots for clinical correlation analysis of SPP1 at different clinical T-stages. *P < 0.05.

To explore the association between OERDEGs (CDKN3, PINK1, SPP1, TFRC) and ESCA development, we generated ROC curves for these OERDEGs in the TCGA-ESCA dataset, with clinical status (ESCA vs. Normal) as the outcome variable (Fig. 8A-D). Among these, the AUCs for CDKN3, SPP1, and TFRC were greater than 0.9, demonstrating outstanding sensitivity and specificity for predicting ESCA. The AUC for PINK1 was greater than 0.8 and less than 0.9, indicating high sensitivity and specificity for predicting ESCA. Furthermore, ESCA patients were stratified into N0 & N1 and N2 & N3 subgroups based on N-stage, and ROC curves for OERDEGs were generated using this grouping as the outcome variable (Fig. 8E). The AUC for CDKN3 was between 0.6 and 0.7, suggesting relatively lower accuracy in predicting N-stage. Similarly, patients with ESCA were grouped into T1 & T2 and T3 & T4 subgroups based on T-stage, and ROC curves were constructed accordingly (Fig. 8F). SPP1's AUC was above 0.6 but below 0.7, signifying a relatively lower level of accuracy in predicting T-stage. Additionally, we performed time-dependent ROC analysis to evaluate the predictive capability of OERDEGs for survival in ESCA patients (Fig. 8G, 8H). The AUCs exceeded 0.5 for 1-, 2-, and 3-year survival, indicating that CDKN3 and SPP1 could effectively predict the outcome of ESCA patients. Finally, we analyzed OERDEGs in subgroups based on progression-free interval (PFI) and clinical T-stage, respectively. The results indicated that the expression of CDKN3 was associated with PFI (P < 0.05), while the expression of SPP1 correlated with T-stage (P < 0.05) (Fig. 8I, 8J).

### Cox analysis

**Figure 9:**
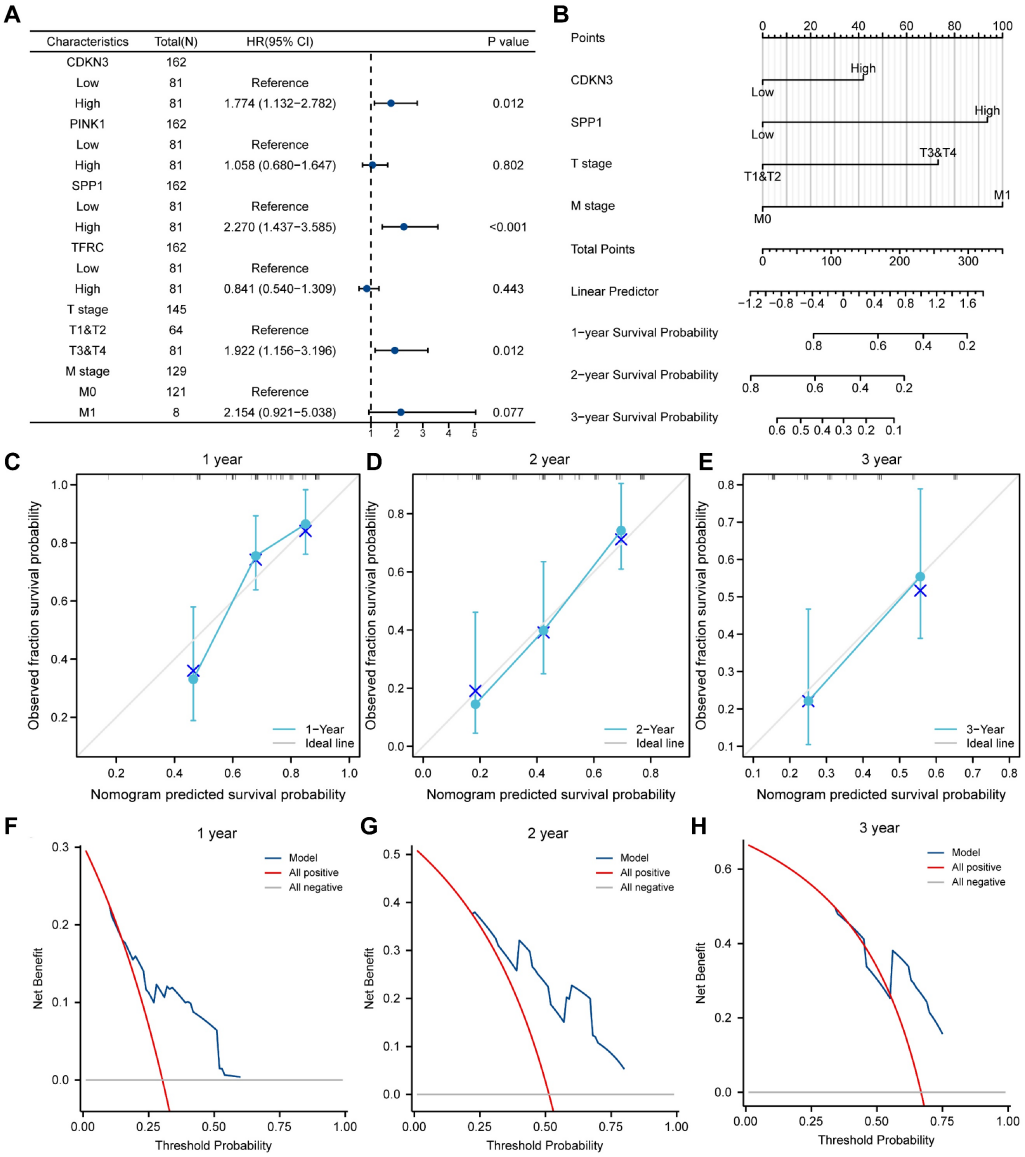
** Cox regression model. A-B** Forest plots (A) and column line plots (B) depicting multifactor Cox regression analysis for OERDEGs. **C-E** 1-year (C), 2-year (D), and 3-year (E) calibration plots for the nomogram analysis of the multifactor Cox regression model. **F-H** 1-year (F), 2-year (G), and 3-year (H) DCA plots for the LASSO-Cox regression prognostic model.

To validate the established LASSO model, we investigated the association between the expression of OERDEGs (CDKN3, PINK1, SPP1, TFRC) and prognosis using univariate and multivariate Cox regression analysis in the TCGA-ESCA dataset. The Cox regression model incorporated T-stage and M-stage, and the forest plots illustrated a significant association between the expression of CDKN3 and SPP1 and prognosis (Fig. 9A, Table S13). Next, we conducted nomogram analysis to evaluate the predictive performance of the multivariate Cox regression model (Fig. 9B). We performed calibration analysis on the nomogram plots for 1-, 2-, and 3-year predictions (Fig. 9C-E). According to the calibration plots, the blue curves for 2- and 3-year predictions closely matched the grey ideal curve, suggesting that the prediction accuracy of the 2- and 3-year models was superior to that of the 1-year model. Furthermore, we evaluated the clinical utility of the LASSO-Cox regression prognostic model for 1-, 2-, and 3-year predictions through DCA (Fig. 9F-H). The blue line representing the model consistently outperformed the red line for all positive cases and the grey line for all negative cases. The range of x values was the widest for 2-year predictions, indicating that the results for 2-year predictions were more reliable.

### Detection of OERDEGs in ESCA tissues and their prognostic implications

**Figure 10:**
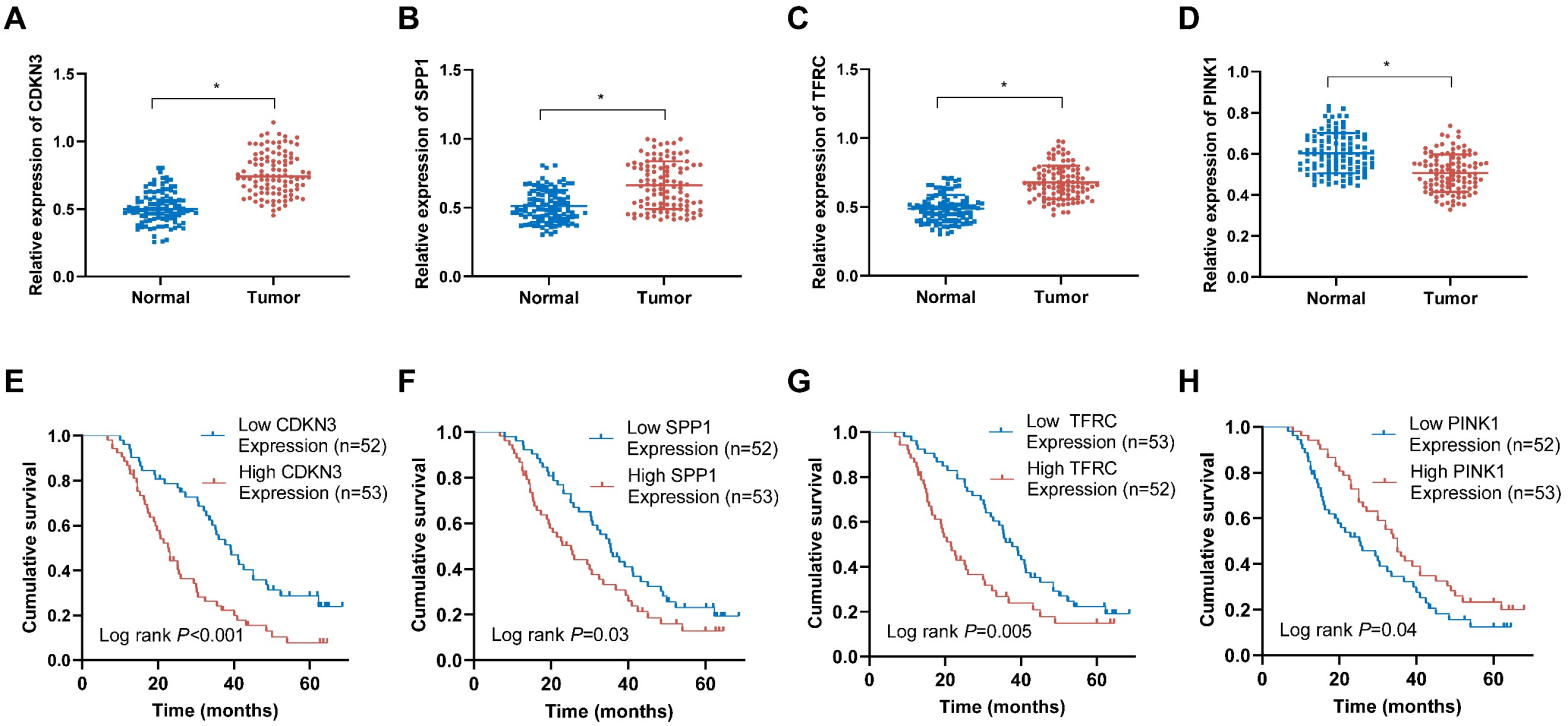
** The expression levels of OERDEGs in ESCA tissues and their correlation with prognosis. A-D** The expression levels of CDKN3, SPP1, TFRC and PINK1 were determined in 105 pairs of ESCA tissues and adjacent normal tissues using qRT-PCR method. **E-H** The Kaplan-Meier curves were generated to examine the correlation between the expression levels of CDKN3, SPP1, TFRC and PINK1 in ESCA tissues and the prognosis of ESCA patients. * P <0.05.

To evaluate the expression patterns of the OERDEGs (CDKN3, SPP1, TFRC, and PINK1) in ESCA tissues, qRT-PCR assays were performed. The analysis demonstrated significant upregulation of CDKN3, SPP1, and TFRC in ESCA tissues compared to those in corresponding normal tissues (Fig. 10A-C). Conversely, PINK1 expression was notably downregulated in ESCA tissues (Fig. 10D). Based on the median expression levels of these genes, ESCA tissues were stratified into high-expression and low-expression groups. As shown in Fig. 10E-H, elevated levels of CDKN3, SPP1, and TFRC were associated with poorer prognosis, whereas reduced expression of PINK1 was linked to adverse prognostic outcomes. Among these OERDEGs, the role of TFRC in ESCA has not been previously reported. Therefore, TFRC was selected for further investigation to explore its biological function in ESCA. By analyzing TFRC expression in 105 ESCA tissue samples and correlating these levels with clinical pathological parameters and patient prognosis, we found that higher expression of TFRC was significantly associated with TNM stage, tumor invasion depth, and lymph node metastasis (P < 0.05) (Table S14).

### The expression of TFRC is elevated in ESCA cells under both ROS and ER stress conditions

**Figure 11:**
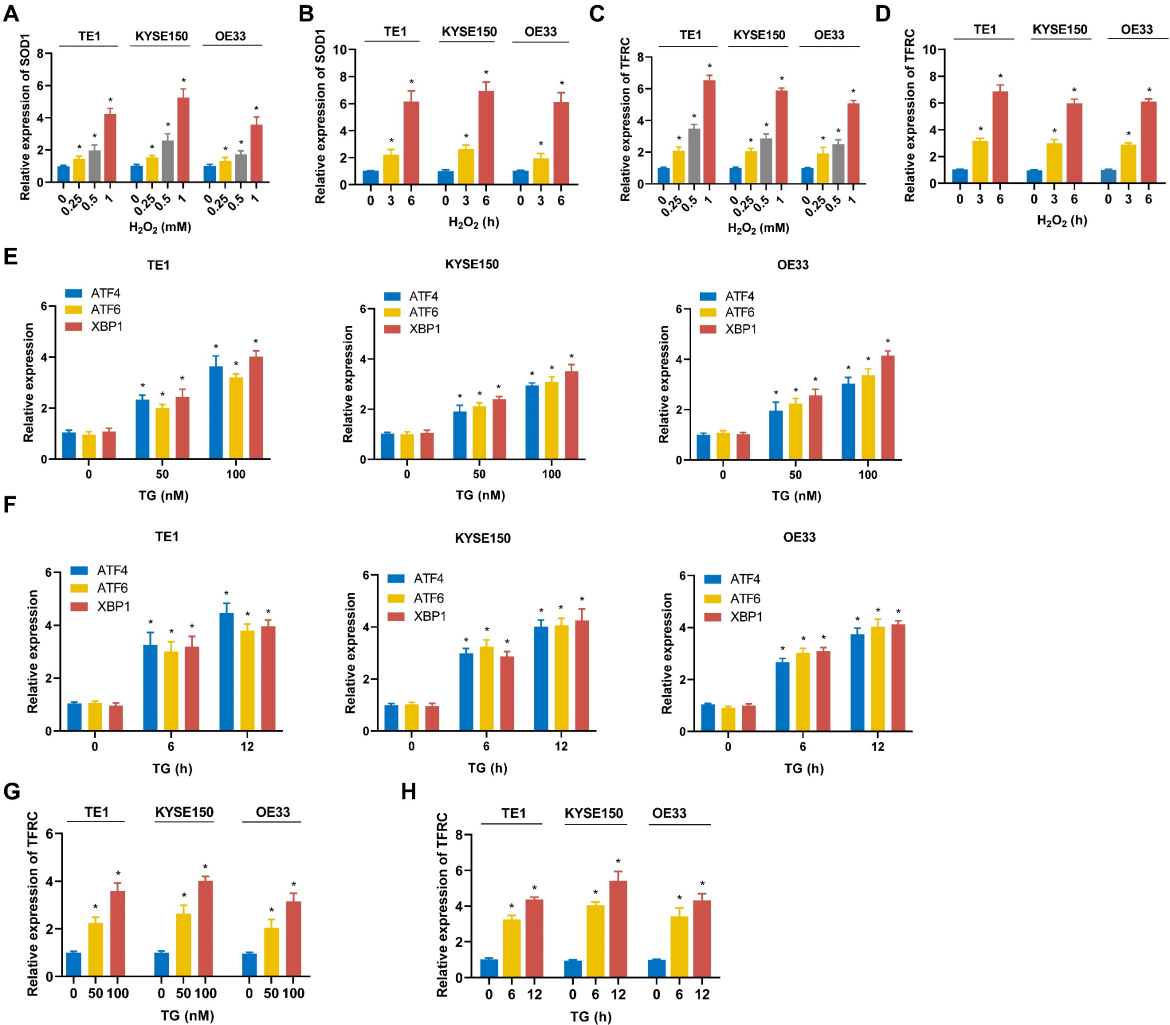
**Expression of TFRC in ESCA cells under oxidative and ER stress conditions. A** TE1, KYSE150 and OE33 cells were treated with H_2_O_2_ for 3 hours, and SOD1 mRNA levels were measured using qRT-PCR. **B** After exposure to 0.5 mM H_2_O_2_ for varying durations, the expression of SOD1 in TE1, KYSE150, and OE33 cells was analyzed by qRT-PCR. **C** TE1, KYSE150, and OE33 cells were treated with H_2_O_2_ for 3 hours, and the mRNA expression of TFRC was assessed via qRT-PCR. **D** The expression levels of TFRC were quantified using qRT-PCR after treating TE1, KYSE150, and OE33 cells with 0.5 mM H_2_O_2_ for different periods of time. **E** TE1, KYSE150 and OE33 cells were treated with various concentrations of TG for 12 hours, and the expression levels of genes related to the ER stress pathway were measured using qRT-PCR. **F** ESCA cells were exposed to 100nM TG for different durations, followed by qRT-PCR analysis to evaluate the expression of ER stress-related genes. **G** The mRNA expression levels of TFRC in TE1, KYSE150, and OE33 cells were analyzed after a 12-hour treatment with specific concentrations of TG, using qRT-PCR. **H** After treating ESCA cells with 100 nM TG for varying time intervals, TFRC expression was assessed by qRT-PCR. Data are presented as the mean ± SD from at least three independent experiments. *P < 0.05.

To model oxidative stress, ESCC cell lines (TE1, KYSE150) and EAC cell lines (OE33) were treated with hydrogen peroxide (H_2_O_2_). As shown in Fig. 11A and 11B, H_2_O_2_ treatment resulted in upregulation of the ROS-related gene SOD1 in all cell lines, indicating that these cells underwent significant oxidative stress. Furthermore, the mRNA expression of TFRC was also elevated in each cell line following H_2_O_2_ exposure (Fig. 11C, 11D). Notably, TFRC upregulation exhibited both time- and dose-dependent patterns. These findings suggest that ROS stress may induce TFRC mRNA expression. To model ER stress, TE1, KYSE150, and OE33 were treated with thapsigargin (TG). The mRNA expression levels of ER stress markers XBP1, ATF4, and ATF6 were quantified using qRT-PCR, and all three genes were significantly upregulated, confirming the successful establishment of the ER stress model (Fig. 11E, 11F). Furthermore, qRT-PCR analysis demonstrated a marked increase in TFRC expression in ESCA cells following TG-induced ER stress, indicating that TFRC acted as a response gene to ER stress (Fig. 11G, 11H).

### TFRC enhances ESCA cell proliferation, migration, and invasion

**Figure 12:**
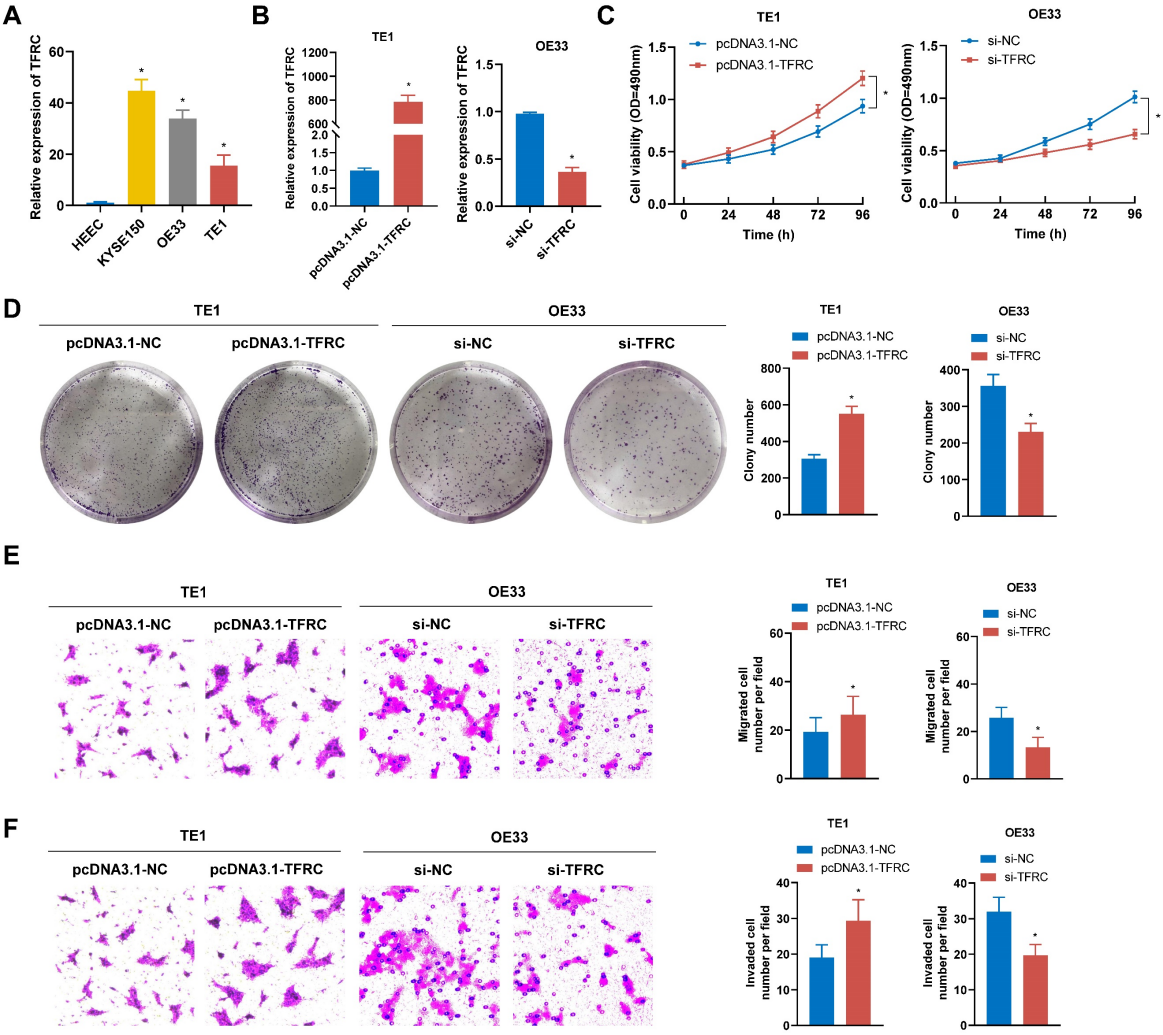
** TFRC promotes the proliferation, migration, and invasion of ESCA cells. A** TFRC expression levels were analyzed in ESCA cells and HEEC using qRT-PCR. **B** The transfection efficiency of TFRC overexpression in TE1 cells and knockdown in OE33 cells was confirmed by qRT-PCR. **C-D** Cell proliferation was evaluated in TFRC-overexpressing TE1 cells and TFRC-knockdown OE33 cells using MTS assays (C) and colony formation assays (D). **E-F** The migration and invasion capabilities of TFRC-overexpressing TE1 cells and TFRC-knockdown OE33 cells were assessed through transwell migration (E) and invasion (F) assays. Data are presented as the mean ± SD from at least three independent experiments. *P < 0.05.

To assess the malignant biological role of TFRC in ESCA, a series of functional assays were performed. qRT-PCR analysis revealed a marked increase in TFRC mRNA expression levels in ESCA cell lines compared to the normal esophageal epithelial cell line (Fig. 12A). The pcDNA3.1-TFRC plasmid was used to overexpress TFRC in TE1 cells, while siRNA was used to knock down TFRC in OE33 cells. The efficiency of transfection was verified by qRT-PCR (Fig. 12B). MTS and colony formation assays showed that TFRC significantly enhanced ESCA cell proliferation (Fig. 12C, 12D). Additionally, transwell migration and invasion assays showed that TFRC promoted the migration and invasion of ESCA cells (Fig. 12E, 12F). These findings indicate that TFRC promotes the malignant behaviors of ESCA cells in vitro.

### TFRC affects the HIF-1α and NOTCH1 signaling pathway in ESCA cells

**Figure 13:**
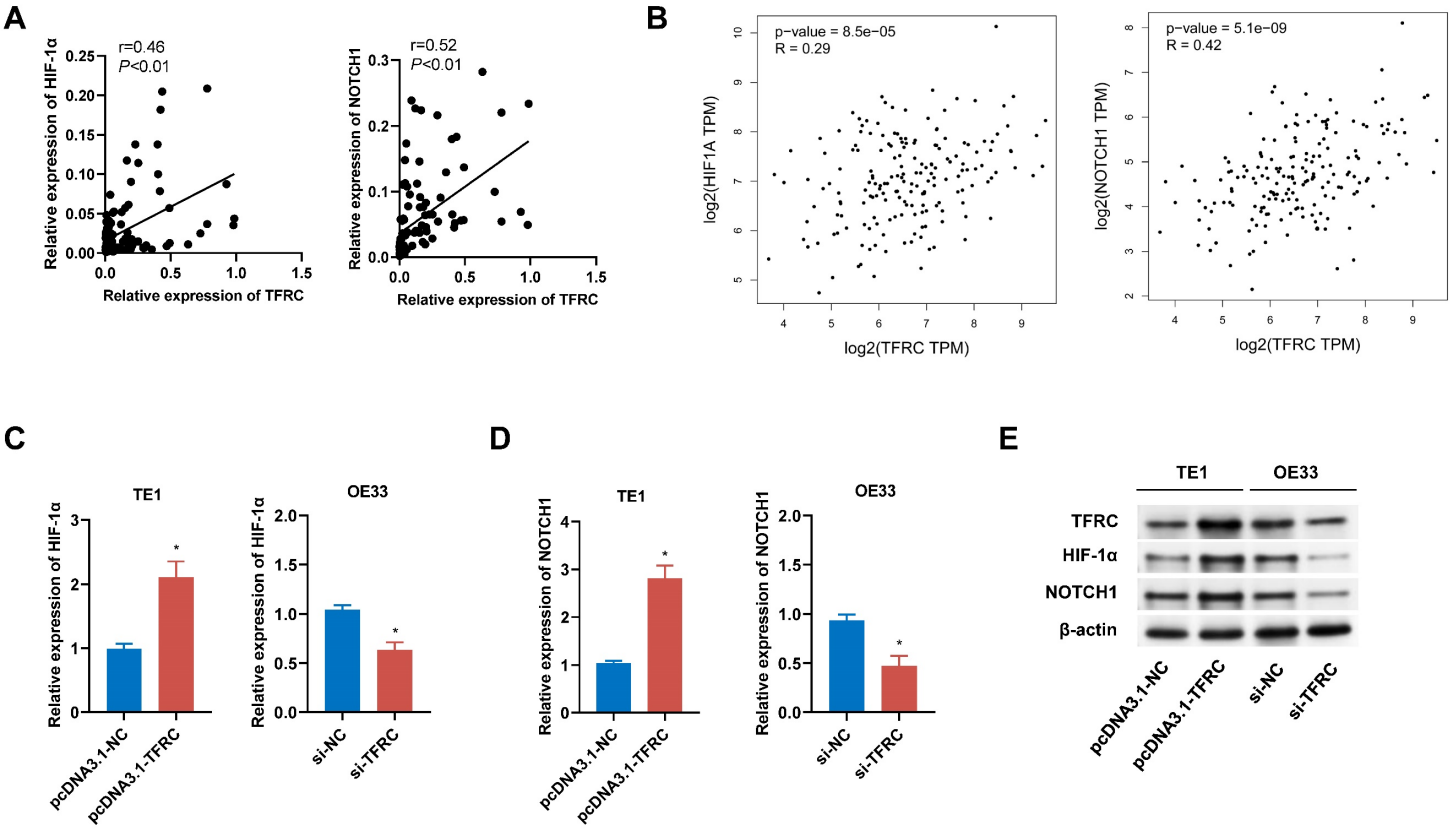
** The impact of TFRC on HIF-1α and NOTCH1 signaling pathways in ESCA cells. A** Correlation analysis between TFRC expression and the mRNA levels of HIF-1α and NOTCH1 in ESCA tissues (n = 105). **B** Relative mRNA expression levels of HIF-1α and NOTCH1 in relation to TFRC expression, based on GEPIA database analysis. **C-D** qRT-PCR analysis showing the relative mRNA expression levels of HIF-1α (C) and NOTCH1 (D) in TE1 cells with TFRC upregulation and OE33 cells with TFRC downregulation. **E** Western blot analysis demonstrating the protein levels of HIF-1α and NOTCH1 in response to TFRC overexpression and knockdown.

To validate the signaling pathways identified through bioinformatics analysis, we conducted both qRT-PCR and Western blot analyses. Correlation analysis of 105 paired ESCA tissue samples revealed a positive relationship between TFRC expression and the expression levels of HIF-1α and NOTCH1, which was further corroborated by data from the GEPIA database (Fig. 13A, 13B). qRT-PCR analysis demonstrated that upregulation of TFRC in TE1 cells led to increased mRNA levels of HIF-1α and NOTCH1, while downregulation of TFRC in OE33 cells resulted in reduced mRNA levels of these genes (Fig. 13C, 13D). Consistently, Western blot experiments confirmed that TFRC overexpression significantly elevated the protein levels of HIF-1α and NOTCH1, whereas TFRC knockdown led to decreased protein expression of these targets (Fig. 13E).

### TFRC promotes the proliferation, migration, and invasion of ESCA cells by regulating the HIF-1α and NOTCH1 signaling pathway

**Figure 14:**
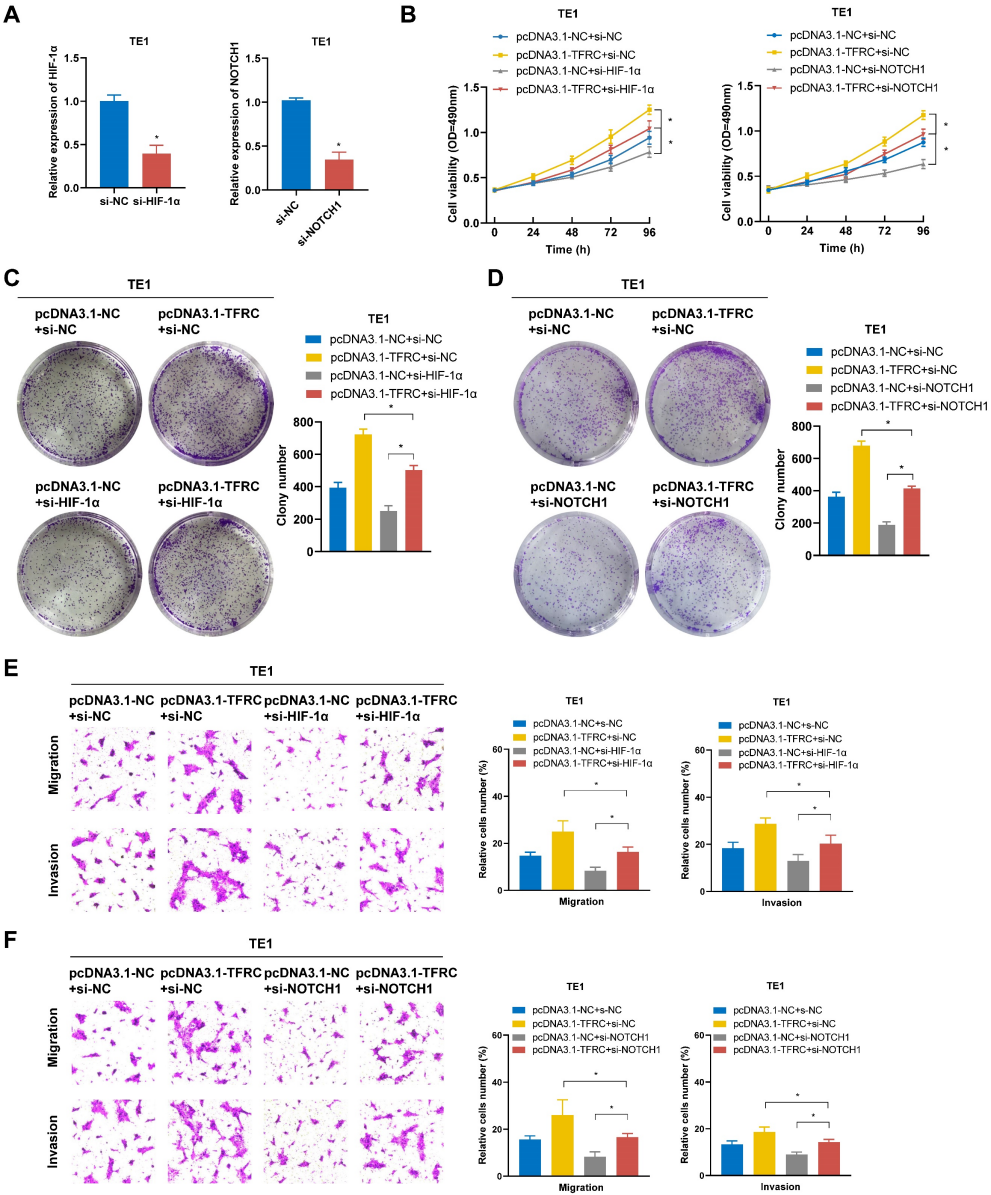
** TFRC promotes the proliferation, migration, and invasion of ESCA cells via the HIF-1α and NOTCH1 signaling pathway. A** The transfection efficiency of HIF-1α and NOTCH1 knockdown in TE1 cells was confirmed by qRT-PCR. B The effect of HIF-1α and NOTCH1 knockdown on the proliferative capacity of TFRC-overexpressing TE1 cells was evaluated using MTS assays. **C-D** Colony formation assays were used to assess the impact of HIF-1α (C) and NOTCH1 (D) knockdown on the proliferation of TFRC-overexpressing TE1 cells. **E-F** Transwell migration and invasion assays were performed to evaluate the effects of silencing HIF-1α (E) and NOTCH1 (F) on cell migration and invasion in TFRC-overexpressing TE1 cells. Data are presented as the mean ± SD from at least three independent experiments. *P < 0.05.

To verify whether TFRC exerts oncogenic effects in ESCA cells by regulating the HIF-1α and NOTCH1 pathway, rescue experiments were conducted through the knockdown of HIF-1α and NOTCH1 in TFRC-overexpressing TE1 cells. The transfection efficiency of si-HIF-1α and si-NOTCH1 in TE1 cells was verified using qRT-PCR (Fig. 14A). As shown in Fig. 14B-D, MTS and colony formation assays demonstrated that silencing HIF-1α and NOTCH1 partially reversed the increased cell proliferation resulting from TFRC overexpression. Similarly, Transwell migration and invasion assays showed that knocking down HIF-1α and NOTCH1 also alleviated the increased cell migration and invasion resulting from TFRC overexpression (Fig. 14E, 14F). Collectively, these findings suggest that TFRC exerts oncogenic effects in ESCA by regulating the HIF-1α and NOTCH1 pathway.

## Discussion

ESCA often presents with subtle symptoms in its early stages, leading most patients to be diagnosed at an advanced stage, which limits treatment options and results in poor prognosis 22. Despite the growing understanding of the biological mechanisms underlying ESCA in recent years, the lack of reliable early diagnostic biomarkers and therapeutic targets continues to present significant challenges for clinical management.

Oxidative stress and ER stress have emerged as critical contributors to cancer development and progression. Tumors often exhibit elevated levels of ROS and activation of the UPR, both of which contribute to carcinogenesis through genetic mutations and dysregulation of key signaling pathways 23-25. ROS drive tumor progression by promoting malignancy-associated traits, including enhanced proliferation, resistance to apoptosis, and EMT 26, 27. The UPR also plays a crucial role in regulating key processes in tumor biology, including cell proliferation, angiogenesis, and therapy resistance across various cancer types 28-30. Moreover, oxidative stress and ER stress often coexist and interact in pathological conditions 15, 31. ROS can modulate ER stress and activate the UPR, while the UPR can stimulate ROS production within the ER lumen, creating a feedback loop that contributes to cellular dysfunction 32, 33.

Studies have shown that both oxidative stress and ER stress play significant roles in the development and progression of ESCA. For example, ROS upregulate MMP-2/9 through activation of the NF-κB pathway, thereby enhancing the invasiveness of ESCA cells 34. Additionally, ROS facilitate the methylation of the p16 promoter, leading to reduced p16 expression and increased cell proliferation, which contributes to the malignant transformation of ESCA 35. Furthermore, TMTC3 activates the PERK pathway, leading to the nuclear translocation of ATF4, which induces EMT and accelerates tumor cell growth and metastasis 36. However, despite these studies highlighting the roles of oxidative stress and ER stress in ESCA, many underlying mechanisms remain poorly understood, particularly concerning how these stress responses interact and drive tumor progression.

In this study, 12 OERDEGs were identified through bioinformatics analysis of TCGA and GEO datasets. Pathway enrichment analyses, including GSEA and GSVA, revealed significant associations with apoptosis, the PI3K-AKT-mTOR signaling pathway, fatty acid metabolism, glycolysis, the NOTCH signaling pathway, and the HIF-1 signaling pathway. These findings underscore the importance of stress responses in ESCA. Such integrative analyses can provide valuable insights for future research and help elucidate how environmental factors influence the biological behavior of ESCA. Additionally, a prognostic model was constructed using LASSO regression analysis and validated through ROC curves and Cox regression analysis. This model identified CDKN3, PINK1, SPP1, and TFRC as significant prognostic biomarkers for ESCA. The strong correlation between the expression levels of these genes and patient prognosis suggests that they could serve as reliable indicators for assessing ESCA progression and treatment response. Incorporating these biomarkers into clinical evaluations may enhance risk stratification and facilitate the development of personalized treatment strategies for ESCA patients.

Among these four biomarkers, TFRC (also known as TFR1) has garnered particular attention due to its crucial biological role in cancer. TFRC is regulated by various stimuli, including intracellular iron concentration, inflammation, and oxidative stress. For instance, hypoxia induces TFRC gene transcription by enabling HIFs to bind to specific promoter elements 37. TFRC is highly expressed in multiple cancers and influences cell proliferation, migration, invasion, and apoptosis by modulating metabolism, inflammation, and iron homeostasis 38-40. Targeting TFRC has shown promising antitumor effects, as demonstrated by Shimosaki et al., who developed a human IgG monoclonal antibody against TFR1, significantly suppressing the growth of HTLV-1-associated adult T-cell leukemia/lymphoma cells 41. Thus, TFRC presents a compelling molecular target for cancer therapy, yet its role in ESCA has not been fully established.

Our findings reveal that TFRC is upregulated in response to oxidative and ER stress, correlating with poorer prognosis in ESCA patients. TFRC promotes the proliferation, migration, and invasion of ESCA cells by regulating HIF-1α and NOTCH1. Both HIF-1α and NOTCH1 pathways are known to regulate hypoxia responses, angiogenesis, and cancer stem cell renewal, making them key drivers of aggressive cancer phenotypes 42-44. Therefore, through the synergistic action of these two pathways, TFRC may serve as a potential therapeutic target for ESCA.

However, this study has several limitations. In vitro methods restrict the direct applicability of the current findings to clinical practice. Future studies should validate these results using animal models or culture systems that better mimic the tumor microenvironment, to gain a more comprehensive understanding of the role of OERDEGs in ESCA progression and treatment response. Additionally, the interactions and networks among these OERDEGs may reveal potential synergistic effects and pathways that drive ESCA progression. Further investigation into how these genes cooperate or compete could provide insights into their collective roles in tumor biology, potentially leading to the identification of new therapeutic targets. Moreover, potential therapies targeting TFRC and other OERDEGs need to be further validated in preclinical models. Such studies will help assess their safety and efficacy, offering potential new treatment options for ESCA.

In conclusion, this study identified several OERDEGs as potential prognostic biomarkers through bioinformatics analysis, providing important insights into the interplay between oxidative stress, ER stress, and ESCA progression. By highlighting the interactions between stress responses and key cellular signaling pathways, such as HIF-1α and NOTCH1, this research underscores the significance of stress responses in cancer biology. These findings open up new avenues for the early diagnosis and treatment of ESCA, with TFRC, in particular, emerging as a promising therapeutic target with significant clinical translation potential.

## Supplementary Material

Supplementary tables.
