# Supplementary material for: Identification and Characterization of Oxidative Stress and Endoplasmic Reticulum Stress-Related Genes in Esophageal Cancer
Source: J Cancer. 2025 Mar 21;16(7):2103–23. doi: 10.7150/jca.104376 (PMC12036101; doi:10.7150/jca.104376)
Supplement: Supplementary file 1 — Supplementary tables. [file jcav16p2103s1.pdf]

Table S1. List of gene symbols of OERGs

Gene Symbols							
SIGMAR1	RACK1	PPARGC1A	PARK7	LRRK2	PARP1	CTNNB1	NQO1
EDN1	EDNRA	EGFR	EIF2S1	AIF1	AKT1	ERN1	FABP1
FOXO1	FOXO3	SIRT1	FOS	ALOX5	ABL1	G6PD	PRDX5
GBA	GJB2	HTRA2	GPR37	GPX1	GSR	UBQLN1	HIF1A
HMOX1	APEX1	HSF1	HSPA1A	HSPA1B	HSPB1	APOA4	IL6
IL10	INS	JUN	MAPT	MCL1	MAP3K5	MGST1	MMP2
MMP9	MPO	ATF4	NFE2L1	NFE2L2	NOS3	ATP2A2	P4HB
PRDX1	PRKN	PKD2	PML	ANKZF1	PRKAA1	PRKAA2	PRKCD
SELENOS	MAPK1	MAPK3	MAPK8	MAPK9	SELENON	PXN	BCL2
RELA	PINK1	SNCA	SOD1	SOD2	SRC	STAU1	TLR4
TNF	TP53	TXN	WNT1	ERMP1	ZC3H12A	SESN2	MAP1LC3A
CAT	BECN1	AIFM1	CD36	KEAP1	CDK1	PRDX4	TXNIP
COL1A1	CRYAB	CYP2E1	DHCR24	TOR1A	GSTP1	ERO1A	HP
APOE	APP	PON2	PPP2CB	PRNP	PSEN1	PTGS1	PTGS2
BAK1	SELENOK	SLC8A1	SP1	CAPN2	CASP3	PPP1R15B	STC2
ADIPOQ	NR1H2	AKAP9	TRAF2	STIP1	HLA-A	RUNX2	PDIA3
TNFSF11	DYSF	CAMK2G	HERPUD1	ALOX15	TRPV4	ABCC8	UBC
FMR1	HSPA6	PTGIS	UBQLN2	HYOU1	SREBF1	SERPINH1	VCL
CASP4	TNFRSF10B	LPL	VAPB	ELAVL1	CDH2	DSP	BCL2L11
F8	DNAJB1	ITIH4	DES	INSR	DNAH8	FCGR2B	MYH6
FKBP5	FKRP	NLRP3	QDPR	CASP2	THBS1	CEBPB	PPP1R15A
SIL1	PSEN2	PDIA2	PTPN1	EP300	SGK1	MFN2	TIA1
CD4	CFTR	CTSB	EIF4G1	EIF2AK1	ATXN3	HLA-B	ATF3
CXCR4	NLRP1	KCNE1	ENO2	GJA1	HMGCR	HSP90AB1	CCR6
DSPP	VIM	ANK2	MAP2K7	CR2	ELN	BMP2	TNFSF10
DMD	CST3	STUB1	APOB	TLR9	APAF1	TGM2	HSD17B10
RB1	KCNQ1	ITGB1	SERPINA1	PIK3R1	CDC42	BRCA1	IREB2
MAP2	CTSD	CACNB4	IGF2R	CASP7	EIF2AK2	COL2A1	IAPP
PRKCA	TTR	EIF4E	CRYAA	G3BP1	SCN4A	OXT	OPA1
CRHR1	POR	TGFB3	BACE1	DRD5	SERPINA3	HSPA9	SCN2A
ITPR1	MT-TK	DNM1L	PLA2G6	KCNH2	NPPB	H6PD	VDAC1
ADRB2	NR3C2	GSK3B	MYC	PTK2	HLA-DRA	UGT1A1	CYB5R3
GPT	TFRC	MAP2K1	CD40	BCL2L1	EGR1	EEF1A1	ATXN2
PTEN	ELANE	POMC	FUS	FASLG	HLA-DRB1	TF	HFE
ACP1	NOTCH1	PTPN11	AGTR1	HSP90B1	FN1	CACNA1S	HTT
CDK5	CALR	TTN	APOA1	CCND1	MAPKAPK2	NPY	HSPD1
LEP	RAC1	SNAP25	EPO	RPS27A	MDM2	NGF	CREB1
ATF6	TLR2	ATM	CDKN1A	RHOA	PPARA	KDR	SPP1
HMGB1	CLU	PIK3CG	CDKN3	SERPINE1	AGER	PRL	LDLR
TRDN	CASQ2	CALM1	SHC1	COMT	ANXA5	TH	STAT3
EIF2AK3	TNFRSF1A	TERT	SLC2A1	FAS	CYP1A2	NR3C1	HSPA8
C9orf72	GFAP	IGF1	EGF	MYH7	CYP1A1	NOX4	CASP9
F2	PPARG	KNG1	MTOR	BAX	VCAM1	CACNA1C	CASP8
LMNA	XBP1	CRH	CAV1	TARDBP	MAPK10	ACE	ESR1
VCP	ICAM1	CP	SCN5A	BDNF	NFKB1	CAV3	TYR
GSTM1	DDIT3	SQSTM1	HSPA5	VWF	GAPDH	VEGFA	RYR2

CCL2	RYR1	CYBB	HSPA4	IFNG	HSP90AA1	TGFB1	CYBA
CXCL8	CRP	ALB	SLC6A4	IL1B	CYCS	PON1	XDH
MAPK14	CPT2	NOS1	NOS2				

Table S2. Primer sequences

Gene	Primer sequences (5'–3')
GAPDH	Forward: GAAGGTGAAGGTCGGAGTC
	Reverse: GAAGATGGTGATGGGATTTC
CDKN3	Forward: AGCCGCCCAGTTCAATACAA
	Reverse: CCTGGAAGAGCACATAAACCG
SPP1	Forward: GAAGTTTCGCAGACCTGACAT
	Reverse: GTATGCACCATTCAACTCCTCG
TFRC	Forward: ATTGAACCTGGACTATGAGAG
	Reverse: TGGAAGTAGCACGGAAGA
PINK1	Forward: TCTCAAGTCCGACAACATCCT
	Reverse: ATTGCCACCACGCTCTACA
SOD1	Forward: GCCGATGTGTCTATTGAA
	Reverse: AGCGTTTCCTGTCTTTGT
ATF4	Forward: GGTTCTCCAGCGACAAGG
	Reverse: TCTCCAACATCCAATCTGTCC
ATF6	Forward: AACTTTCCGTGACTAAACCTGT
	Reverse: CCTTTAATCTCGCCTCTAACCC
XBP1	Forward: CAGCTCAGACTGCCAGAGATCGAA
	Reverse: TCCGCCTCCTCTTCAGCAACCA
HIF-1α	Forward: CCAGTTACGTTCCTTCGATCAG
	Reverse: GTAGTGGTGGCATTAGCAGTAG
NOTCH1	Forward: GGACGTCAGACTTGGCTCAG
	Reverse: ACATCTTGGGACGCATCTGG

Table S3. Antibodies used for Western blot assays

Antibody	Company	Catalogue number
β-Actin	ZenBioScience	380624
TFRC	ZenBioScience	381603
HIF-1α	ZenBioScience	340462
NOTCH1	ZenBioScience	380355

Table S4. List of gene symbols of OERDEGs

Gene symbols				
CDK1	CDKN3	COL1A1	CXCL8	MMP9
PINK1	SERPINE1	SERPINH1	SLC2A1	SPP1

TFRC	VCAM1	PTGS1

Table S5. List of expression differences of OERDEGs

Gene Symbol	log2FoldChange	p-value	p. adj
CDK1	2.02563646	3.15E-17	1.42E-14
CDKN3	2.3513379	1.39E-18	9.06E-16
COL1A1	2.50712827	1.52E-07	5.81E-06
CXCL8	4.32559781	1.98E-16	6.96E-14
MMP9	3.40661793	3.57E-10	2.81E-08
PINK1	-1.0099608	5.88E-06	0.00012502
PTGS1	-1.1489447	0.00651446	0.03381348
SERPINE1	2.09898089	7.04E-05	0.00096832
SERPINH1	1.80795125	3.77E-10	2.94E-08
SLC2A1	2.69253937	1.31E-08	6.93E-07
SPP1	5.3759968	1.12E-16	4.21E-14
TFRC	1.99744593	1.17E-09	8.10E-08
VCAM1	1.10325786	0.00321719	0.01982329

Table S6. GO/KEGG enrichment analysis results of OERDEGs

ONTOLOGY	ID	Description	GeneRatio	BgRatio	p-value	p.adjust	q-value
BP	GO:0030198	extracellular matrix organization	6/12	368/18670	4.71e-08	4.85e-05	2.36e-05
BP	GO:0043062	extracellular structure organization	6/12	422/18670	1.06e-07	5.46e-05	2.66e-05
BP	GO:0000302	response to reactive oxygen species	4/12	232/18670	1.06e-05	0.004	0.002
BP	GO:1903862	positive regulation of oxidative phosphorylation	2/12	10/18670	1.70e-05	0.004	0.002
BP	GO:0046677	response to antibiotic	4/12	327/18670	4.09e-05	0.007	0.003
CC	GO:0062023	collagen-containing extracellular matrix	4/12	406/19717	7.69e-05	0.007	0.004
CC	GO:0005788	endoplasmic reticulum lumen	3/12	309/19717	7.55e-04	0.034	0.021
CC	GO:0042470	melanosome	2/12	106/19717	0.002	0.041	0.025
CC	GO:0048770	pigment granule	2/12	106/19717	0.002	0.041	0.025
CC	GO:0042383	sarcolemma	2/12	136/19717	0.003	0.052	0.032
MF	GO:0002020	protease binding	3/12	128/17697	7.75e-05	0.005	0.002
MF	GO:0061134	peptidase regulator activity	3/12	219/17697	3.79e-04	0.012	0.005
MF	GO:0005518	collagen binding	2/12	67/17697	9.09e-04	0.014	0.005
MF	GO:0001618	virus receptor activity	2/12	74/17697	0.001	0.014	0.005

MF	GO:0104005	hijacked molecular function	2/12	74/17697	0.001	0.014	0.005
KEGG	hsa04933	AGE-RAGE signaling pathway in diabetic complications	4/10	100/8076	4.39e-06	3.29e-04	2.40e-04
KEGG	hsa04066	HIF-1 signaling pathway	3/10	109/8076	2.68e-04	0.010	0.007
KEGG	hsa04218	Cellular senescence	3/10	156/8076	7.68e-04	0.019	0.014
KEGG	hsa05219	Bladder cancer	2/10	41/8076	0.001	0.021	0.015
KEGG	hsa05144	Malaria	2/10	50/8076	0.002	0.025	0.018

Table S7. GSEA analysis of the TCGA-ESCA dataset

Description	setSize	enrichmentScore	NES	p-value	p.adjust	q-values
REACTOME_DNA_DAMAGE_TELOMERE_STRESS_INDUCED_SENESCENCE	80	0.73744104	2.72373873	0.00180505	0.01304095	0.00902064
REACTOME_PRC2_METHYLATES_HISTONES_AND_DNA	71	0.69852879	2.54217173	0.00182482	0.01304095	0.00902064
REACTOME_SENESCENCE_ASSOCIATED_SECRETORY_PHENOTYPE_SASP_	110	0.65356211	2.52014442	0.00180832	0.01304095	0.00902064
REACTOME_HDACS_DEACETYLATE_HISTONES	91	0.66724896	2.51762951	0.00182482	0.01304095	0.00902064
REACTOME_CELLULAR_SENESCENCE	194	0.59549157	2.49360086	0.00164745	0.01304095	0.00902064
REACTOME_HCMV_INFECTION	159	0.61374723	2.4901183	0.0017094	0.01304095	0.00902064
REACTOME_OXIDATIVE_STRESS_INDUCED_SENESCENCE	122	0.61550059	2.418152	0.00174216	0.01304095	0.00902064
REACTOME_GENE_SILENCING_BY_RNA	136	0.60103049	2.39245331	0.0017331	0.01304095	0.00902064
REACTOME_INTERLEUKIN_10_SIGNALING	45	0.70985204	2.36160988	0.00191939	0.01304095	0.00902064
WP_PHOTODYNAMIC_THERAPYINDUCED_NFKB_SURVIVAL_SIGNALING	35	0.74769938	2.35165958	0.00192308	0.01304095	0.00902064
REACTOME_TCF_DEPENDENT_SIGNALING_IN_RESPONSE_TO_WNT	231	0.53355042	2.27303845	0.00162866	0.01304095	0.00902064
PID_IL23_PATHWAY	37	0.70842208	2.2708253	0.00187266	0.01304095	0.00902064
REACTOME_RMTS_METHYLATE_HISTONE_ARGININES	76	0.61481516	2.25862728	0.00184843	0.01304095	0.00902064
REACTOME_INTERLEUKIN_4_AND_INTERLEUKIN_13_SIGNALING	111	0.58344604	2.25236477	0.00181159	0.01304095	0.00902064
REACTOME_AUF1_HNRNP_D0_BINDS_AND_DESTABILIZES_MRNA	55	0.64223161	2.23988397	0.0018622	0.01304095	0.00902064
REACTOME_HATS_ACETYLATE_HISTONES	139	0.56040806	2.2347975	0.00174825	0.01304095	0.00902064
REACTOME_STABILIZATION_OF_P53	57	0.62211557	2.20072746	0.00180832	0.01304095	0.00902064
REACTOME_DECTIN_1_MEDIATED_NONCANONICAL_NF_KB_SIGNALING	62	0.61026214	2.18098717	0.00178891	0.01304095	0.00902064
REACTOME_METABOLISM_OF_POLYAMINES	59	0.60627819	2.14534799	0.00182482	0.01304095	0.00902064
REACTOME_PRE_NOTCH_EXPRESSION_AND_PROCESSING	118	0.54873271	2.14442753	0.00175439	0.01304095	0.00902064

Table S8. GSVA of the TCGA-ESCA dataset

ID	logFC	AveExpr	t	P.Value	P. adj.	B
HALLMARK_E2F_TARGETS	0.64533307	0.00065017	5.65274133	5.72E-08	2.48E-06	7.89501104
HALLMARK_G2M_CHECKPOINT	0.57097633	0.00140977	5.54154778	9.92E-08	2.48E-06	7.36695794
HALLMARK_BILE_ACID_METABOLISM	-0.3840152	-0.0001741	-5.2577695	3.91E-07	6.51E-06	6.0538299
HALLMARK_MYC_TARGETS_V1	0.5306426	0.00233643	4.80019694	3.20E-06	4.01E-05	4.04608105
HALLMARK_DNA_REPAIR	0.37306835	-0.0170058	4.6003791	7.70E-06	7.70E-05	3.21390964
HALLMARK_MYC_TARGETS_V2	0.53444311	-0.0029183	4.34714325	2.25E-05	0.00018724	2.20002089

HALLMARK_KRAS_SIGNALING_DN	-0.3238048	-0.0046782	-4.21351	3.88E-05	0.00027745	1.68386536
HALLMARK_MITOTIC_SPINDLE	0.30215716	-0.0128773	3.90774626	0.00012953	0.00069603	0.55346012
HALLMARK_MTORC1_SIGNALING	0.33671904	-0.007894	3.89486062	0.00013607	0.00069603	0.50739877
HALLMARK_UNFOLDED_PROTEIN_RESPONSE	0.3149531	0.00276347	3.88888542	0.00013921	0.00069603	0.48608353
HALLMARK_INTERFERON_ALPHA_RESPONSE	0.39771069	0.00346612	3.40079985	0.00081919	0.0037236	-1.1592777
HALLMARK_FATTY_ACID_METABOLISM	-0.267686	0.00117008	-3.3611827	0.00093845	0.00391021	-1.284354
HALLMARK_ESTROGEN_RESPONSE_EARLY	-0.2497546	-0.0005093	-3.3374465	0.00101747	0.00391336	-1.3586703
HALLMARK_UV_RESPONSE_UP	0.23054538	-0.0038078	3.27899909	0.00123929	0.00442604	-1.539669
HALLMARK_XENOBIOTIC_METABOLISM	-0.2296009	0.01419187	-3.2284017	0.00146686	0.00470819	-1.6940551
HALLMARK_UV_RESPONSE_DN	-0.2591284	0.00401489	-3.2203194	0.00150662	0.00470819	-1.7185177
HALLMARK_SPERMATOGENESIS	0.23384455	0.00096414	3.13243602	0.00200847	0.00560016	-1.9809602
HALLMARK_ANDROGEN_RESPONSE	-0.2358593	0.00089583	-3.1312714	0.00201606	0.00560016	-1.9843942
HALLMARK_PI3K_AKT_MTOR_SIGNALING	0.22336863	-0.0058916	3.09357816	0.00227632	0.00576899	-2.0949163
HALLMARK_HEME_METABOLISM	-0.2063673	-0.0026332	-3.0893204	0.0023076	0.00576899	-2.1073249
HALLMARK_MYOGENESIS	-0.2614267	-0.0021476	-3.0611753	0.0025245	0.00601072	-2.1889604
HALLMARK_ADIPOGENESIS	-0.2281892	-0.0074251	-3.0378809	0.00271807	0.00617744	-2.2560149
HALLMARK_NOTCH_SIGNALING	0.24004192	-0.0155674	2.95403874	0.00353347	0.00768145	-2.4935117
HALLMARK_APOPTOSIS	0.20767484	0.00230802	2.8108193	0.00546024	0.01137551	-2.8851594
HALLMARK_INTERFERON_GAMMA_RESPONSE	0.28771787	0.00750707	2.77041929	0.00615517	0.01231034	-2.9924071
HALLMARK_PEROXISOME	-0.1953483	-0.0063351	-2.6362898	0.00907583	0.01745352	-3.3381835
HALLMARK_TGF_BETA_SIGNALING	0.21603451	0.00212297	2.45542399	0.01497239	0.02680552	-3.7791481
HALLMARK_GLYCOLYSIS	0.18346378	-0.003386	2.45446576	0.01501109	0.02680552	-3.7814063
HALLMARK_ESTROGEN_RESPONSE_LATE	-0.1744409	0.0007029	-2.35924	0.01932872	0.03332538	-4.0016882
HALLMARK_WNT_BETA_CATENIN_SIGNALING	0.19436398	-0.0015724	2.19545877	0.0293438	0.04890634	-4.3612954
HALLMARK_ALLOGRAFT_REJECTION	0.21471797	0.00492428	2.10027824	0.037026	0.05971935	-4.5589911

Table S9. OERDEGs in PPI network ranked by MCC method

Rank	Name	Score
1	VCAM1	49
2	SPP1	48
2	MMP9	48
2	SERPINE1	48
5	COL1A1	25
6	CXCL8	24
7	TFRC	2
7	SERPINH1	2
7	SLC2A1	2
10	CDK1	1

10	CDKN3	1

Table S10. mRNA-drug network of OERDEGs

mRNA		drug
CDKN3	-	RONICICLIB
CDKN3	-	AZD-5438
CDKN3	-	AT-7519
CDKN3	-	PHA-793887
MMP9	-	MARIMASTAT
MMP9	-	PRINOMASTAT
MMP9	-	ANDECALIXIMAB
MMP9	-	S-3304
MMP9	-	CURCUMIN PYRAZOLE
MMP9	-	TOZULERISTIDE
MMP9	-	CURCUMIN
MMP9	-	INCYCLINIDE
MMP9	-	BEVACIZUMAB
MMP9	-	CARBOXYLATED GLUCOSAMINE
MMP9	-	DEMETHYLWEDELOLACTONE
MMP9	-	CELECOXIB
SERPINE1	-	ALEPLASININ
SERPINE1	-	UROKINASE
SERPINE1	-	CITALOPRAM
SERPINE1	-	URIDINE
SERPINE1	-	REGRAMOSTIM
SERPINE1	-	PREMARIN
SERPINE1	-	EPIRUBICIN
SERPINE1	-	ORLISTAT
SERPINE1	-	DIAPLASININ
SERPINE1	-	DALTEPARIN
SERPINE1	-	LEVOTHYROXINE
SERPINE1	-	FLUOXETINE
SERPINE1	-	VASOPRESSIN
SERPINE1	-	DEFIBROTIDE
SERPINE1	-	NIMODIPINE
SERPINE1	-	DEXAMETHASONE
SERPINE1	-	HYDROCHLOROTHIAZIDE
SERPINE1	-	TIPLASININ

SERPINE1	-	CETRORELIX
SERPINE1	-	CAPTOPRIL
COL1A1	-	OCRIPLASMIN
COL1A1	-	COLLAGENASE CLOSTRIDIUM HISTOLYTICUM
SLC2A1	-	PHENYTOIN
SLC2A1	-	THYMIDINE
SLC2A1	-	TRIAMCINOLONE
SLC2A1	-	PROGESTERONE
SLC2A1	-	THIOCTIC ACID
SLC2A1	-	GENTAMICIN
SLC2A1	-	PIOGLITAZONE
SLC2A1	-	ROSIGLITAZONE
SLC2A1	-	GENISTEIN
SLC2A1	-	GLUFOSFAMIDE
SLC2A1	-	DIAZEPAM
SPP1	-	ASK-8007
SPP1	-	CALCITONIN
SPP1	-	ALTEPLASE
SPP1	-	WORTMANNIN
SPP1	-	GENTAMICIN
SPP1	-	TACROLIMUS
VCAM1	-	TROGLITAZONE
VCAM1	-	MERCAPTOPURINE
VCAM1	-	DEXAMETHASONE
VCAM1	-	CYCLOSPORINE
CXCL8	-	ABX-IL8
CXCL8	-	HUMAX-IL8
CXCL8	-	LEFLUNOMIDE
CXCL8	-	YANGONIN
CXCL8	-	E319
CXCL8	-	FOSCARNET
CXCL8	-	NAPROXEN
CXCL8	-	ALDRIN
CXCL8	-	COLCHICINE
CXCL8	-	MIDAZOLAM
CXCL8	-	FENTANYL
CXCL8	-	ACETAMINOPHEN
CXCL8	-	CORONOPILIN
CXCL8	-	DIPYRIDAMOLE
CXCL8	-	IBUPROFEN

CXCL8	-	IONOMYCIN
CXCL8	-	CHLORDANE
CXCL8	-	DANAZOL
CXCL8	-	CHEMBL1902074
CXCL8	-	OMEPRAZOLE
CXCL8	-	DINITRO CRESOL
CXCL8	-	QUESTIOMYCIN B
CXCL8	-	FENRETINIDE
CXCL8	-	HEPTACHLOR
CXCL8	-	PYROGALLOL
CXCL8	-	CANERTINIB
CXCL8	-	HYDROQUINONE
CXCL8	-	ENDOSULFAN
CXCL8	-	EMODIN
CXCL8	-	LANSOPRAZOLE
CXCL8	-	RETINAL
CXCL8	-	HARMINE HYDROCHLORIDE
CXCL8	-	PACLITAXEL
CXCL8	-	BEVACIZUMAB
CXCL8	-	PAMIDRONIC ACID
CXCL8	-	TALC
CXCL8	-	TRETINOIN
CXCL8	-	SUNITINIB
CXCL8	-	CETUXIMAB
CXCL8	-	CHEMBL1579130
CXCL8	-	ALPRAZOLAM
CXCL8	-	METHIMAZOLE
CXCL8	-	RETINOL
CXCL8	-	RIBAVIRIN
CXCL8	-	TERFENADINE
CXCL8	-	DICYCLOHEXYLCARBODIIMIDE
CXCL8	-	CEFTRIAXONE
CXCL8	-	ASPIRIN
CXCL8	-	CLARITHROMYCIN
CXCL8	-	DACARBAZINE
CXCL8	-	PENTOXIFYLLINE
CXCL8	-	CIDOFOVIR
CXCL8	-	BROXURIDINE
CXCL8	-	TROGLITAZONE
CXCL8	-	DICHLORVOS

CXCL8	-	VERAPAMIL
CDK1	-	AG-24322
CDK1	-	RONICICLIB
CDK1	-	AT-7519
CDK1	-	DINACICLIB
CDK1	-	ZOTIRACICLIB
CDK1	-	ALVOCIDIB
CDK1	-	CHIR-99021
CDK1	-	RIVICICLIB
CDK1	-	SELICICLIB
CDK1	-	ALSTERPAULLONE
CDK1	-	PHA-793887
CDK1	-	RGB-286638
CDK1	-	MILCICLIB
CDK1	-	AZD-5438
CDK1	-	RG-547
CDK1	-	KENPAULLONE
CDK1	-	CLOFIBRATE
CDK1	-	SP-600125
CDK1	-	CHEMBL1082552
CDK1	-	FENOFIBRATE
CDK1	-	SOTRASTAURIN
CDK1	-	GO-6976
CDK1	-	LAUROGUADINE
CDK1	-	BMS-345541
CDK1	-	PATULIN
CDK1	-	SERTRALINE
CDK1	-	GW441756X
CDK1	-	CHEMBL403183
CDK1	-	CENISERTIB
CDK1	-	ARUNCIN B
CDK1	-	LY-2090314
CDK1	-	PROTUBOXEPIN A
CDK1	-	CHEMBL541400
CDK1	-	AZD-1080
CDK1	-	CHEMBL225519
CDK1	-	R-406
CDK1	-	CYC-116
CDK1	-	ROTENONE
CDK1	-	SNS-314

CDK1	-	PF-00562271
CDK1	-	CLOTRIMAZOLE
CDK1	-	WITHAFERIN A
CDK1	-	CINNARIZINE
CDK1	-	RG-1530
CDK1	-	CHEMBL578061
CDK1	-	(RS)-ROSCOVITINE
CDK1	-	RUCAPARIB
CDK1	-	TAE-684

Table S11. mRNA-TF network of OERDEGs

mRNA		TF
CDK1	-	BRD4
CDK1	-	CDK9
CDK1	-	CREBBP
CDK1	-	E2F1
CDK1	-	EHF
CDK1	-	ERG
CDK1	-	ETS1
CDK1	-	FLI1
CDK1	-	FOS
CDK1	-	FOXM1
CDK1	-	GABPA
CDK1	-	HDAC1
CDK1	-	MAZ
CDK1	-	NR2F2
CDK1	-	SPI1
CDK1	-	USF1
CDKN3	-	BRD4
CDKN3	-	E2F1
CDKN3	-	EBF1
CDKN3	-	FOS
CDKN3	-	FOXM1
CDKN3	-	GABPA
CDKN3	-	GATA1
CDKN3	-	HDAC1
CDKN3	-	KMT2A
CDKN3	-	MAX

CDKN3	-	MAZ
CDKN3	-	PBX3
CDKN3	-	TFAP2C
CDKN3	-	ZEB1
CDKN3	-	ZNF384
COL1A1	-	E2F1
COL1A1	-	E2F6
COL1A1	-	EBF1
COL1A1	-	EGR1
COL1A1	-	ELF1
COL1A1	-	ARID3A
COL1A1	-	EP300
COL1A1	-	ERG
COL1A1	-	ETS1
COL1A1	-	ARNT
COL1A1	-	FLI1
COL1A1	-	FOSL1
COL1A1	-	FOSL2
COL1A1	-	FOXA1
COL1A1	-	FOXA2
COL1A1	-	GABPA
COL1A1	-	GATA1
COL1A1	-	GTF2I
COL1A1	-	HDAC2
COL1A1	-	IRF1
COL1A1	-	IRF4
COL1A1	-	JUN
COL1A1	-	BCL3
COL1A1	-	KLF1
COL1A1	-	KLF4
COL1A1	-	KLF5
COL1A1	-	KLF9
COL1A1	-	LMNB1
COL1A1	-	MAX
COL1A1	-	MAZ
COL1A1	-	BHLHE40
COL1A1	-	MED12
COL1A1	-	MEF2A
COL1A1	-	MXI1
COL1A1	-	MYB

COL1A1	-	MYC
COL1A1	-	MYOD1
COL1A1	-	NFIC
COL1A1	-	NR2F2
COL1A1	-	BRD2
COL1A1	-	BRD4
COL1A1	-	PBX3
COL1A1	-	POLR2A
COL1A1	-	PPARG
COL1A1	-	RAD21
COL1A1	-	CBX3
COL1A1	-	RNF2
COL1A1	-	RUNX1T1
COL1A1	-	RUNX2
COL1A1	-	RUNX3
COL1A1	-	SMC1A
COL1A1	-	SMC3
COL1A1	-	SP1
COL1A1	-	SP4
COL1A1	-	SPI1
COL1A1	-	SRF
COL1A1	-	STAG1
COL1A1	-	SUMO2
COL1A1	-	CDK8
COL1A1	-	CDK9
COL1A1	-	TCF12
COL1A1	-	TCF3
COL1A1	-	TCF7L2
COL1A1	-	TEAD4
COL1A1	-	CEBPA
COL1A1	-	TFAP2A
COL1A1	-	TFAP2C
COL1A1	-	TFAP4
COL1A1	-	CEBPB
COL1A1	-	USF1
COL1A1	-	USF2
COL1A1	-	VEZF1
COL1A1	-	CHD1
COL1A1	-	ZBTB7A
COL1A1	-	ZC3H8

COL1A1	-	ZEB1
COL1A1	-	ZNF263
COL1A1	-	CREB1
COL1A1	-	CREBBP
COL1A1	-	CTCF
COL1A1	-	CTCFL
COL1A1	-	ZNF92
CXCL8	-	AR
CXCL8	-	BRD4
CXCL8	-	CEBPA
CXCL8	-	CEBPB
CXCL8	-	EP300
CXCL8	-	ERG
CXCL8	-	ETS1
CXCL8	-	FLI1
CXCL8	-	FOSL1
CXCL8	-	FOXA1
CXCL8	-	FOXA2
CXCL8	-	GABPA
CXCL8	-	GATA1
CXCL8	-	JUN
CXCL8	-	JUND
MMP9	-	EP300
MMP9	-	ETS1
MMP9	-	FLI1
MMP9	-	MAFF
MMP9	-	MAZ
MMP9	-	MYB
MMP9	-	NFE2
MMP9	-	NR2F2
MMP9	-	POLR2A
MMP9	-	RUNX1
MMP9	-	RUNX1T1
MMP9	-	SMARCB1
MMP9	-	SMC1A
MMP9	-	SPI1
MMP9	-	TCF12
MMP9	-	TFAP2A
MMP9	-	TFAP2C
MMP9	-	CREB1

MMP9	-	CREBBP
PINK1	-	BRCA1
PINK1	-	BRD2
PINK1	-	CTCF
PINK1	-	E2F6
PINK1	-	EP300
PINK1	-	HDAC1
PINK1	-	HNF4A
PINK1	-	HNF4G
PINK1	-	KLF4
PINK1	-	MAX
PINK1	-	MAZ
PINK1	-	MNT
PINK1	-	MXI1
PINK1	-	POLR2A
PINK1	-	RUNX1T1
PINK1	-	RUNX2
PINK1	-	TFAP2C
PINK1	-	TP63
PINK1	-	USF1
SERPINE1	-	E2F6
SERPINE1	-	EGR1
SERPINE1	-	ELF1
SERPINE1	-	ELK4
SERPINE1	-	EP300
SERPINE1	-	ERG
SERPINE1	-	ETS1
SERPINE1	-	ETV1
SERPINE1	-	FLI1
SERPINE1	-	FOXA1
SERPINE1	-	FOXA2
SERPINE1	-	ARNT
SERPINE1	-	FOXP2
SERPINE1	-	GABPA
SERPINE1	-	ATF3
SERPINE1	-	HDAC1
SERPINE1	-	KLF1
SERPINE1	-	KLF4
SERPINE1	-	MAX
SERPINE1	-	MAZ

SERPINE1	-	MXI1
SERPINE1	-	MYB
SERPINE1	-	MYBL2
SERPINE1	-	MYC
SERPINE1	-	MYOD1
SERPINE1	-	BHLHE40
SERPINE1	-	NR2F2
SERPINE1	-	NRIP1
SERPINE1	-	PHF8
SERPINE1	-	POLR2A
SERPINE1	-	BRD4
SERPINE1	-	RFX5
SERPINE1	-	RXRA
SERPINE1	-	SIN3A
SERPINE1	-	SMARCA4
SERPINE1	-	SP1
SERPINE1	-	SPI1
SERPINE1	-	STAT1
SERPINE1	-	TBP
SERPINE1	-	TEAD4
SERPINE1	-	TFAP2A
SERPINE1	-	TFAP2C
SERPINE1	-	TFAP4
SERPINE1	-	TP63
SERPINE1	-	USF1
SERPINE1	-	USF2
SERPINE1	-	WDR5
SERPINE1	-	CEBPA
SERPINE1	-	CEBPB
SERPINE1	-	ZEB1
SERPINE1	-	ZNF263
SERPINE1	-	ZNF384
SERPINE1	-	CTBP2
SERPINE1	-	CTCF
SERPINH1	-	BRD2
SERPINH1	-	CREB1
SERPINH1	-	CTCF
SERPINH1	-	EBF1
SERPINH1	-	ELF1
SERPINH1	-	ELK3

SERPINH1	-	ERG
SERPINH1	-	ETS1
SERPINH1	-	FLI1
SERPINH1	-	GABPA
SERPINH1	-	GTF2B
SERPINH1	-	GTF2I
SERPINH1	-	KDM1A
SERPINH1	-	KLF9
SERPINH1	-	MAZ
SERPINH1	-	NFYA
SERPINH1	-	NFYB
SERPINH1	-	NR2F2
SERPINH1	-	PHF8
SERPINH1	-	SMARCA4
SERPINH1	-	SPI1
SERPINH1	-	SRF
SERPINH1	-	TBP
SERPINH1	-	TEAD4
SERPINH1	-	TFAP4
SERPINH1	-	WDR5
SERPINH1	-	ZBTB7A
SERPINH1	-	ZEB1
SLC2A1	-	ARNT
SLC2A1	-	ATF2
SLC2A1	-	ATF3
SLC2A1	-	BACH2
SLC2A1	-	BCL3
SLC2A1	-	BCL6
SLC2A1	-	BHLHE40
SLC2A1	-	BRD2
SLC2A1	-	BRD4
SLC2A1	-	CBX3
SLC2A1	-	CDK8
SLC2A1	-	CDK9
SLC2A1	-	CEBPA
SLC2A1	-	CEBPB
SLC2A1	-	CREB1
SLC2A1	-	CREBBP
SLC2A1	-	CTCF
SLC2A1	-	E2F1

SLC2A1	-	E2F6
SLC2A1	-	EBF1
SLC2A1	-	EGLN2
SLC2A1	-	EHF
SLC2A1	-	EP300
SLC2A1	-	EPAS1
SLC2A1	-	ERG
SLC2A1	-	ESR1
SLC2A1	-	ETS1
SLC2A1	-	ETV1
SLC2A1	-	FLI1
SLC2A1	-	FOSL1
SLC2A1	-	FOSL2
SLC2A1	-	FOXA1
SLC2A1	-	FOXA2
SLC2A1	-	GABPA
SLC2A1	-	GATA1
SLC2A1	-	HDAC1
SLC2A1	-	HDAC2
SLC2A1	-	HNF4G
SLC2A1	-	IRF1
SLC2A1	-	IRF4
SLC2A1	-	JUN
SLC2A1	-	JUNB
SLC2A1	-	JUND
SLC2A1	-	KDM1A
SLC2A1	-	KLF1
SLC2A1	-	KLF3
SLC2A1	-	KLF4
SLC2A1	-	KLF5
SLC2A1	-	KLF9
SLC2A1	-	MAX
SLC2A1	-	MAZ
SLC2A1	-	MECOM
SLC2A1	-	MED12
SLC2A1	-	MXI1
SLC2A1	-	MYB
SLC2A1	-	MYOD1
SLC2A1	-	NFE2
SLC2A1	-	NFIC

SLC2A1	-	NR2F2
SLC2A1	-	PAX6
SLC2A1	-	PBX3
SLC2A1	-	POLR2A
SLC2A1	-	RBPJ
SLC2A1	-	RCOR1
SLC2A1	-	RUNX1T1
SLC2A1	-	SMARCA4
SLC2A1	-	SMARCB1
SLC2A1	-	SMC1A
SLC2A1	-	SMC3
SLC2A1	-	SP1
SLC2A1	-	SPI1
SLC2A1	-	SRF
SLC2A1	-	STAG1
SLC2A1	-	STAT5A
SLC2A1	-	SUMO2
SLC2A1	-	TCF12
SLC2A1	-	TCF3
SLC2A1	-	TCF7L2
SLC2A1	-	TFAP2A
SLC2A1	-	TFAP2C
SLC2A1	-	TFAP4
SLC2A1	-	UBTF
SLC2A1	-	USF1
SLC2A1	-	USF2
SLC2A1	-	YY1
SLC2A1	-	ZBTB17
SLC2A1	-	ZBTB7A
SLC2A1	-	ZNF263
SPP1	-	CEBPA
SPP1	-	FLI1
SPP1	-	SPI1
TFRC	-	E2F1
TFRC	-	E2F6
TFRC	-	EBF1
TFRC	-	EGLN2
TFRC	-	EP300
TFRC	-	EPAS1
TFRC	-	ERG

TFRC	-	ESR1
TFRC	-	ETS1
TFRC	-	ARNT
TFRC	-	FLI1
TFRC	-	FOS
TFRC	-	FOXA1
TFRC	-	GABPA
TFRC	-	GATA1
TFRC	-	ASCL1
TFRC	-	HDAC1
TFRC	-	HDAC2
TFRC	-	HIF1A
TFRC	-	IRF1
TFRC	-	IRF2
TFRC	-	JUN
TFRC	-	KLF1
TFRC	-	KLF4
TFRC	-	KLF5
TFRC	-	LMNB1
TFRC	-	LMO2
TFRC	-	MAX
TFRC	-	MAZ
TFRC	-	BCL6
TFRC	-	BHLHE40
TFRC	-	MYB
TFRC	-	BRD2
TFRC	-	NR2F2
TFRC	-	NRIP1
TFRC	-	BRD4
TFRC	-	PBX1
TFRC	-	RUNX1
TFRC	-	RUNX1T1
TFRC	-	RXRG
TFRC	-	SPI1
TFRC	-	TCF12
TFRC	-	TCF3
TFRC	-	CDK9
TFRC	-	TEAD4
TFRC	-	TFAP2A
TFRC	-	TFAP2C

TFRC	-	CEBPA
TFRC	-	CEBPB
TFRC	-	CEBPD
TFRC	-	WDR5
TFRC	-	ZBTB7A
TFRC	-	ZNF263
TFRC	-	CREB1
TFRC	-	CREBBP
VCAM1	-	CEBPA
VCAM1	-	CEBPB

Table S12. Patient Characteristics of ESCA patients in the TCGA dataset

Characteristic	levels	Overall
n		162
T stage, n (%)	T1	27 (18.6%)
	T2	37 (25.5%)
	T3	77 (53.1%)
	T4	4 (2.8%)
N stage, n (%)	N0	66 (45.8%)
	N1	63 (43.8%)
	N2	9 (6.2%)
	N3	6 (4.2%)
M stage, n (%)	M0	121 (93.8%)
	M1	8 (6.2%)
Gender, n (%)	Female	23 (14.2%)
	Male	139 (85.8%)
Age, n (%)	<=60	83 (51.2%)
	>60	79 (48.8%)
OS event, n (%)	Alive	97 (59.9%)
	Dead	65 (40.1%)
DSS event, n (%)	Alive	115 (71.4%)
	Dead	46 (28.6%)
PFI event, n (%)	Alive	83 (51.2%)
	Dead	79 (48.8%)

Table S13. Cox regression to identify hub genes and clinical features associated with PFI

Characteristics	Total (N)	Univariate analysis		Multivariate analysis	
		Hazard ratio (95% CI)	P value	Hazard ratio (95% CI)	P value
CDKN3	162				
Low	81	Reference			
High	81	1.774 (1.132-2.782)	**0.012**	1.448 (0.838-2.501)	0.185
PINK1	162				

Characteristics	Total (N)	Univariate analysis		Multivariate analysis	
		Hazard ratio (95% CI)	P value	Hazard ratio (95% CI)	P value
Low	81	Reference			
High	81	1.058 (0.680-1.647)	0.802		
SPP1	162				
Low	81	Reference			
High	81	2.270 (1.437-3.585)	**<0.001**	2.285 (1.315-3.971)	**0.003**
TFRC	162				
Low	81	Reference			
High	81	0.841 (0.540-1.309)	0.443		
T stage	145				
T1&T2	64	Reference			
T3&T4	81	1.922 (1.156-3.196)	**0.012**	1.907 (1.113-3.267)	**0.019**
M stage	129				
M0	121	Reference			
M1	8	2.154 (0.921-5.038)	0.077	2.415 (1.018-5.729)	**0.045**

Table S14. Correlation of the expression level of TFRC with various clinicopathologic features of ESCA patients

**Characteristics**	**N**	**TFRC expression**		**P value**
		**Low n (%)**	**High n (%)**	
Age (years)				0.460
< 60	33	14（42.4）	19（57.6）	
≥ 60	72	39（54.2）	33（45.8）	
Gender				0.589
Male	68	33（48.5）	35（51.5）	
Female	37	20（54.1）	17（45.9）	
Pathological differentiation				0.381
Well/Moderate	63	34（54.0）	29（46.0）	
Poor	42	19（45.2）	23（54.8）	
TNM stage				0.002*
I/II	42	29（69.0）	13（31.0）	
III/IV	63	24（38.1）	39（61.9）	
Depth of invasion				0.036*
T1/2	43	27（62.8）	16（37.2）	
T3/4	62	26（41.9）	36（58.1）	
LN metastasis				0.001*
N0	39	28（71.8）	11（28.2）	
N1/2/3	66	25（37.9）	41（62.1）	
Family history of UGIC				
Negative	85	42（49.4）	43（50.6）	0.653
Positive	20	11（55.0）	9（45.0）	

* P < 0.05.
